# Generation of a pseudo-timeline describing progressive human exocrine and endocrine pancreatic pathology in cystic fibrosis through novel semi-quantitative scoring and AI-driven quantitative image analysis

**DOI:** 10.1007/s00125-025-06559-4

**Published:** 2025-10-12

**Authors:** Yara Al-Selwi, Dina Tiniakos, Sarah J. Richardson, Christine S. Flaxman, Lydia Russell, Rowan Coulthard, Rashmi Maheshwari, Nicola Dyson, Minna Honkanen-Scott, Günter Klöppel, James A. M. Shaw, Nicole Kattner

**Affiliations:** 1 Translational and Clinical Research Institute, Newcastle University, Newcastle upon Tyne, UK; 2 Department of Pathology, Aretaieion Hospital, Medical School, National and Kapodistrian University of Athens, Athens, Greece; 3 Islet Biology Group (IBEx), Exeter Centre of Excellence for Diabetes Research (EXCEED), University of Exeter College of Medicine and Health, Exeter, UK; 4 Department of Cellular Pathology, Royal Victoria Infirmary, Newcastle upon Tyne Hospitals NHS Foundation Trust, Newcastle upon Tyne, UK; 5 Department of Pathology, TUM School of Medicine and Health, Technical University Munich, Munich, Germany; 6 Institute of Transplantation, Freeman Hospital, Newcastle upon Tyne Hospitals NHS Foundation Trust, Newcastle upon Tyne, UK

**Keywords:** Artificial intelligence augmented analysis, Cystic fibrosis, Histological scoring, Histopathology, Islets, Pancreas

## Abstract

**Aims/hypothesis:**

Cystic fibrosis (CF) is associated with pancreatic exocrine insufficiency (PEI) early in life and diabetes in at least 50% of adults. Underlying CF-related changes within the pancreas remain incompletely understood due to scarcity of available human tissue, protracted disease course and absence of robust and reproducible analytical approaches. This study aimed to develop and apply a systematic analysis cross-sectionally to CF pancreatic tissue samples to construct a timeline of exocrine and endocrine changes with progressive disease.

**Methods:**

Through a pathologist-led iterative approach, a light-microscopy semi-quantitative scoring system and artificial-intelligence-driven quantitative image analysis for individual pancreatic variables were developed. These were applied to human pancreatic tissue from 29 CF and 58 control donors without pancreatic disease.

**Results:**

Rapid loss of acinar tissue with virtually complete absence and replacement by adipocytes by the age of 7 years was confirmed. Ductal blockage by thickened secretions was associated with increasing ductal dilatation accompanied by periductal fibrosis, followed by ductal loss with involution of associated fibrosis in parallel with increasing adipocyte proportional area. Remaining ducts were relatively small, surrounded by residual fibrosis. Islets became increasingly clustered, initially surrounded by pancreatic stellate cells and fibrosis, then disorganised by interposing fibrotic tissue between endocrine cell regions and surrounded by residual collagen stranding in a ‘lipotic’ pancreas. Overall islet mass was not reduced but proportional beta cell area was reduced from birth without further loss over the course of progressive disease.

**Conclusions/interpretation:**

The natural history of pancreatic CF progresses rapidly from duct blockage and dilatation associated with periductal fibrosis to global fat replacement in keeping with early onset of PEI in the majority of affected individuals. Beta cell proportional area is reduced at birth before clinical evidence of pancreatic endocrine dysfunction without significant further loss of islet/beta cell mass with age. Increasing islet disorganisation and intra-islet collagen deposition in older donors temporo-spatially implicates fibrosis in and around the islet as being aetiologically important in the development of CF-related diabetes.

**Graphical Abstract:**

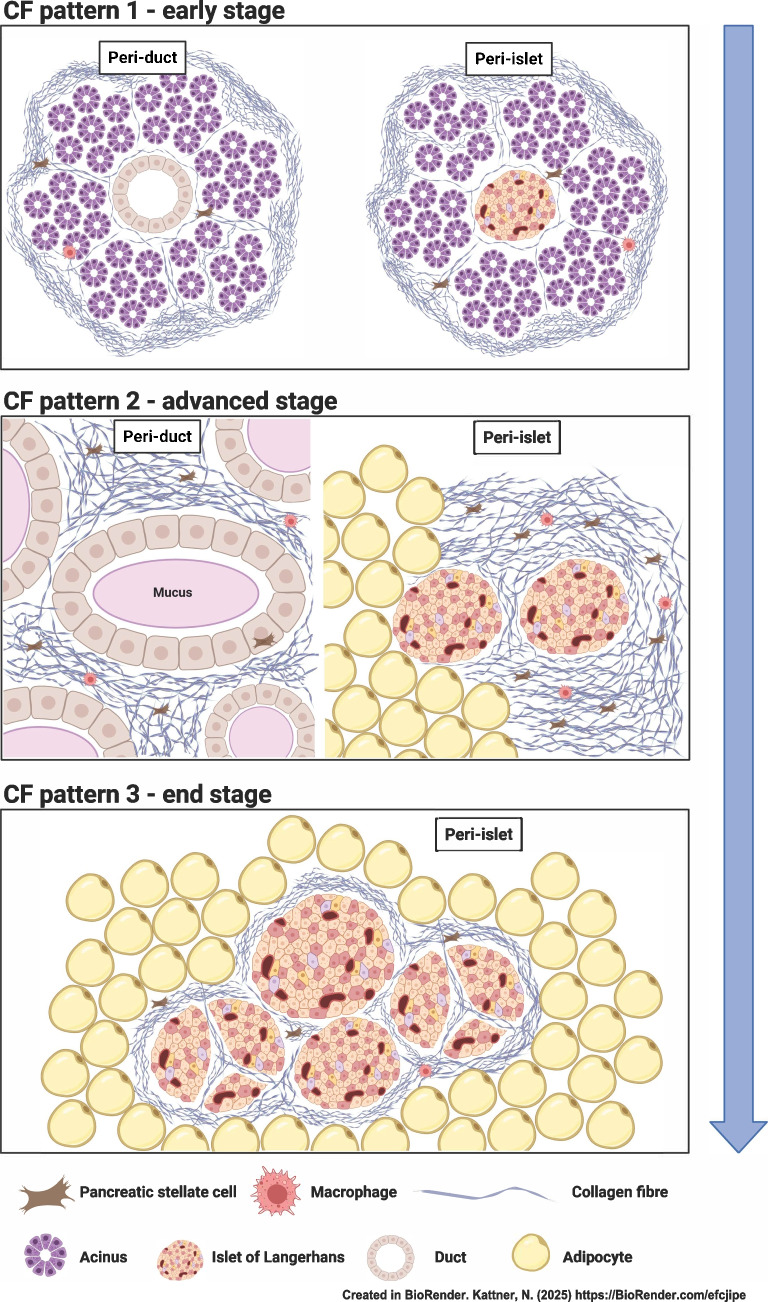

**Supplementary Information:**

The online version contains peer-reviewed but unedited supplementary material available at 10.1007/s00125-025-06559-4.


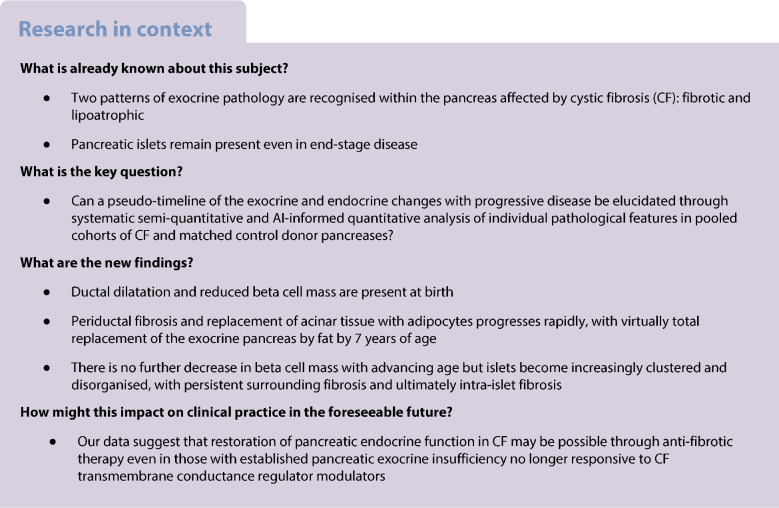


## Introduction

Cystic fibrosis (CF) is associated with pancreatic exocrine insufficiency (PEI) in 80–85% of children [1–3] and CF-related diabetes (CFRD) in at least 50% of adults [4–7]. CFRD adds to treatment burden and is associated with worsened pulmonary function and reduced life expectancy. CFRD is characterised by dysregulated insulin and glucagon secretion [8–11]. While underlying mechanisms remain unclear, it is increasingly accepted that endocrine dysfunction is at least in part mediated by the severe and progredient pancreatic exocrine pathology [12, 13].

CF was named in 1938 due to the post mortem cystic and fibrotic pancreatic pathology in those dying with the condition [14]. Studies of pathological changes within the pancreas have remained largely descriptive, with any quantitative analysis focused on endocrine and immune cells [15–17]. Two distinct pathological patterns have been reported: ‘fibrotic’; and ‘lipoatrophic’ [16, 17]. Reorganisation of the islets originally distributed throughout the acinar parenchyma takes place, leading to irregular islet clustering, although individual islets largely remain identifiable even in end-stage CF [12]. This suggests that islet reorganisation is secondary to *CFTR* mutation-driven ductal plugging initiating tissue remodelling and loss of pancreatic compartmentalisation. Whether pancreatic endocrine pathology is secondary to exocrine disease or intrinsically driven by *CFTR* mutation within the islet remains contentious, however [18]. It is clear that CF pancreatic exocrine pathology progresses incrementally over time but the detailed findings at each stage, exact sequence of events and putative pathomechanisms have not been systematically characterised.

We developed a semi-quantitative scoring system and artificial intelligence (AI)-driven quantification of pathological findings within pancreatic exocrine and endocrine compartments to better define and classify the structural changes. The morphometric tools were applied to pancreatic blocks from donors with CF over a wide age range in comparison with a control cohort to elucidate a ‘pseudo-timeline’ of progressive pathology.

## Methods

### Cohorts

Body of pancreas samples were sourced from several tissue banks to provide a broad age range (premature birth–27 years) in donors with CF (diagnosed clinically and confirmed at post mortem) and optimally matched control donors (birth–29 years). Pancreatic blocks from 19 CF post mortem donors were provided by Exeter Archival Diabetes Biobank (EADB). Blocks from ten CF post mortem donors were provided from the consultation centre archive (endocrine/tumours) (GK, Munich). Available data from CF donors are summarised in electronic supplementary material (ESM) Table 1. Two CF donors had recorded diabetes at time of death, with diabetes status unknown in the remainder (*CFTR* genotype unavailable). Ten control deceased organ donor pancreases without CF/diabetes were provided by the MRC Quality in Organ Donation Whole Pancreas Biobank (QUOD-PANC) in addition to blocks from 46 post mortem donors without CF/diabetes from EADB (ESM Table 2). Post mortem tissue blocks were collected and biobanked by A. Foulis (EADB) and G. Klöppel (Technical University Munich). A single block from each donor was analysed, except for six donors within the Klöppel cohort each providing multiple blocks (ESM Table 1). A subset of Klöppel cohort donors was included in a previous publication [17]. Deceased organ donor biopsies were obtained according to quality-assured Standard Operating Procedures from the anterior body region of the pancreas (Quality in Organ Donation [QUOD] designation: P4A) [19]. Limited tissue availability, loss of tissue integrity during staining and challenges with accurate AI analysis of CF pathology necessitated some variation in numbers of donors or blocks included in each analysis. Available stains used for each donor are listed in ESM Tables 1, 2, with numbers in each analysis cited in figure legends.

QUOD-PANC pancreases were retrieved after informed written donor family consent in compliance with UK Human Tissue Act under UK Human Research Authority ethical approval (05/MRE09/48 and 16NE0230). Use of the EADB archival tissue bank blocks was approved by the West of Scotland Research Ethics Committee (20/WS/0074; IRAS ID: 283620) and use of the Klöppel cohort archival tissue was approved by the local ethics committee of the University Hospital ‘rechts der Isar’, Munich, Germany (281/19 s).

### Staining

Sectioning (4 µm thickness) and staining of the QUOD, EADB and Munich archive tissue was performed by Newcastle NovoPath Laboratories. Histological staining for H&E and chromogenic staining for chromogranin A (CGA), α-smooth muscle actin (SMA), CD45, CD68, CGA/CD31, insulin/pancreatic polypeptide (PP) and glucagon/somatostatin (ESM Table 3) were performed in serial sections using the Ventana Discovery Ultra (Roche Diagnostics, UK) following optimised protocols. Sirius Red Fast Green (SRFG) staining was performed following in-house protocol. Slides were scanned at ×40 magnification at Newcastle Biobank (Leica Aperio AT2; Leica Biosystems [UK]) or in Exeter (PhenoImager Automated Quantitative Pathology Imaging System, Akoya Biosciences, Marlborough, USA). Direct comparison confirmed inter-scanner equivalence (ESM Fig. 1).

### Histological assessment

#### Pattern classification

**Fig. 1:**
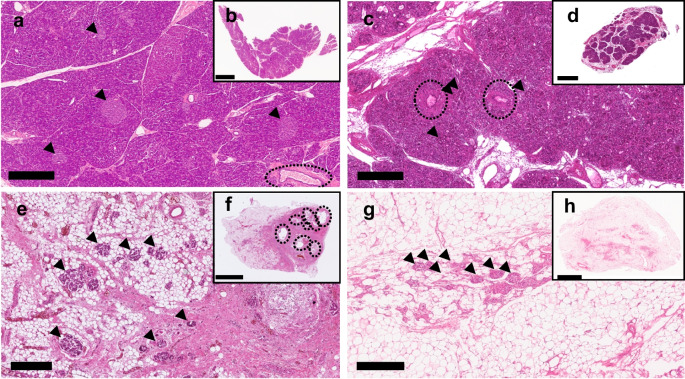
Representative images for control and CF patterns. (**a**, **b**) Control pancreas (donor no. 53) with normal tissue morphology. Lobularity of exocrine parenchyma is preserved with single undilated ducts visible. Islets with normal morphology are distributed within acinar tissue. (**c**, **d**) CF P1 with increased collagen deposition but remaining acinar tissue (CF donor no. 9). Lobularity of exocrine parenchyma is largely preserved with bands of interlobular fibrosis and mild dilatation of small ducts containing inspissated secretions. Islets are embedded in acinar tissue with some irregularity of islet shape. (**e**, **f**) CF P2 with (virtually) complete loss of acinar tissue replaced by collagen and adipocytes (CF donor no. 29). Replacement of acinar parenchyma by fibrosis or fatty tissue leading to extensive loss of exocrine parenchyma. Islets remain present within both fibrotic and lipotic areas, clustered with irregular shape and size. Islets are frequently surrounded and traversed by collagen strands. Ducts and ductules are severely dilated with eosinophilic secretions and mucus in association with extensive periductal fibrosis. (**g**, **h**) CF P3 with total acinar atrophy replaced by fat tissue and clustered islets surrounded by collagen strands (CF donor no. 22). Total loss of acinar tissue and complete replacement by fat tissue is seen. Islets are present but aggregated as irregular clusters in the fat tissue intermingled with collagen strands and fat cells. Extensive duct loss and remaining ducts are surrounded by fibrosis and are mostly small-to-medium in size (ESM Fig. 12). Higher magnification of each tissue section is shown (**b**, **d**, **f**, **h**). Black arrows indicate location of example islets. Dotted circles indicate location of ducts. Scale bar, 500 µm (**a**, **c**, **e**, **g**); 4 mm (**b**, **h**), 3 mm (**d**), 5 mm (**f**). Corresponding CGA- and CD31-stained images are shown in ESM Fig. 2

Each of the 46 tissue blocks from the 29 CF donors was classified (by YAS) a priori into one of three patterns: fibrosis without adipocyte replacement (pattern 1 [P1], ‘fibrotic’); fibrosis with substantial areas of confluent adipocyte replacement (pattern 2 [P2], ‘fibrotic and lipotic’); and global adipocyte replacement without substantial areas of confluent fibrosis (pattern 3 [P3], ‘lipotic’) (Fig. 1 and ESM Fig. 2). Pattern was associated with donor age (*r *= 0.716, *p *< 0.001) (ESM Fig. 3) in keeping with progression over time from fibrosis alone, through mixed fibrotic/lipotic phenotype to virtually total adipocyte replacement.

#### Semi-quantitative scoring

We devised a semi-quantitative scoring system grading (mild, moderate and severe) for individual pathological variables (ESM Table 4). Scoring was undertaken on H&E-stained slides in parallel with SRFG-stained slides for fibrosis evaluation, CGA-stained slides for islet evaluation and CD45-stained slides to evaluate inflammation. All scoring was undertaken by YAS without blinding to donor CF status. Descriptors and difficult cases were discussed until consensus was reached between YAS, DT and GK. A key comprising exemplar images for each variable is shown in ESM Figs 4–9.

#### AI-driven quantitative image analysis

Stained sections were analysed with DenseNet AI V2, Area Quantification modules and Highplex BF modules within the Indica Labs HALO Image analysis platform (Version 3.2.1851.354; Indica Labs, Albuquerque, USA). Quality control was performed and the following areas were excluded from analysis: sections of spleen and intestine; lymph nodes; blurred regions; mucus within large ducts; tissue tears; and areas with non-specific staining. Individual classifiers were prepared for H&E- and SRFG-stained sections. Classifiers were trained on donors with a range of CF pathology and three normal pancreases (donor age <7 days to 3 years). Every example of background, fibrosis, acinar, ducts, islets and fat was annotated manually to ensure accurate training (ESM Fig. 10). Each classifier was trained to 50,000 iterations and until cross-entropy fluctuated between 0.2 and 0.6. Fibrosis area classifiers were trained using SRFG-stained slides with all reported collagen density data derived from SRFG-stained slides. Islets were defined as endocrine cells clusters with area ≥1000 µm^2^. Islet area classifiers were cross-validated in CGA-stained slides confirming correlation between the two methods (ESM Fig. 11). Islet circularity was calculated as (4π×Area)/(2×Perimeter) with a value of ‘1’ equating to a perfect circle. Quantification of individual islet hormone-positive areas was performed using the DenseNet AI V2, Area Quantification and Highplex BF modules within the Indica Labs HALO Image analysis platform (Version 3.2.1851.354) and presented as a percentage of total endocrine area (sum of all hormone-positive areas). Experimenters were not blinded to donor CF status.

### Statistical analyses

Normally distributed data are summarised as mean ± SD with comparisons between CF and control donors undertaken by unpaired Student’s *t* test. For comparison of semi-quantitative scores and AI quantification between tissue blocks with each CF pattern and control samples, linear mixed effect model was used with CF histological pattern set as fixed effect, donor as random effect and Bonferroni post hoc testing. Relationships between two variables were assessed using Pearson and Spearman correlation analysis. Absence of data on donor sex in the EADB cohort precluded any analysis of the potential impact of donor sex on the findings. *p *< 0.05 was considered statistically significant.

## Results

### Semi-quantitative histological scoring

**Fig. 2:**
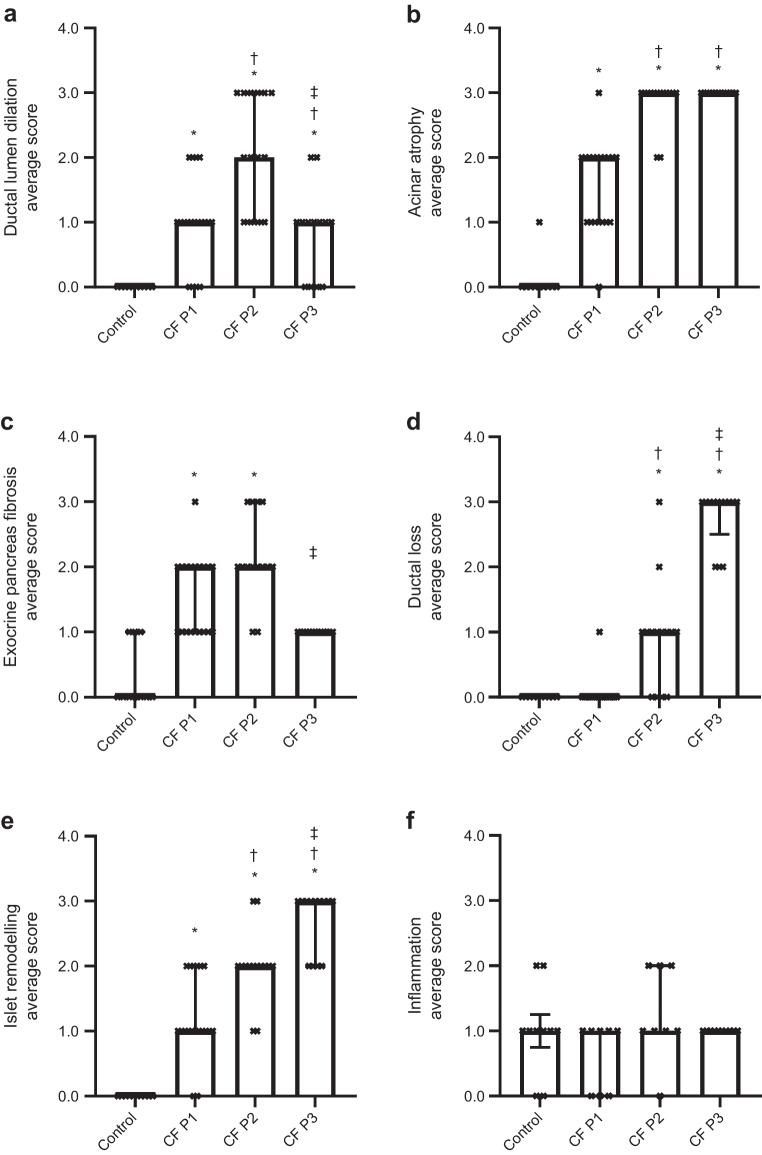
Pattern-based histopathological semi-quantitative analysis to assess control and CF pancreases for individual morphological features: (**a**) ductal lumen dilation; (**b**) acinar atrophy; (**c**) exocrine pancreas fibrosis; (**d**) ductal loss; (**e**) islet remodelling; and (**f**) inflammation. Graphs show median ± IQR. Four of six outcomes were relatively normally distributed and thus all were compared by linear mixed effect model with CF histological pattern set as fixed effect, donor as random effect and Bonferroni post hoc testing. *p < 0.05 vs control;^†^*p *< 0.05 vs CF P1; ^‡^*p *< 0.05 vs CF P2. *n *= 10 control blocks from QUOD deceased organ donors and CF pancreases (*n *= 46 P1 blocks, *n *= 19 P2 blocks, *n *= 14 P3 blocks, *n *= 13 P3 blocks) (**a**–**e**). *n *= 10 control blocks and CF pancreases (*n *= 26 Klöppel cohort blocks alone, *n *= 8 P1 blocks, *n *= 8 P2 blocks, *n *= 10 P3 blocks) (**f**). Scoring was undertaken on slides stained with H&E in parallel with SRFG-stained slides for fibrosis evaluation, CGA-stained slides for islet evaluation and CD45-stained slides to evaluate inflammatory cell infiltration

Following a priori classification of each CF block into one of three pathological patterns (Fig. 1), semi-quantitative scoring for individual pathological features (ESM Table 4) was undertaken and results were compared with those for deceased organ donor pancreases without CF, diabetes or chronic pancreatitis (Fig. 2). Mild ductal dilatation was evident in P1. Duct dilatation was ubiquitous in P2 with identifiable residual ducts in P3 having relatively little dilatation. Exocrine pancreas fibrosis score closely paralleled ductal dilatation score, being significantly greater in P1 than in control donors and peaking in P2. This appeared to be followed by duct loss with no difference in duct loss score for P1 vs control donors, ‘focal’ areas of loss in P2 and ‘severe’ loss in P3. Residual ducts were predominantly small or medium in size and were ubiquitously surrounded by fibrosis even following virtually complete replacement of pancreatic parenchyma by adipocytes (ESM Fig. 12). Acinar atrophy was evident in P1 and virtually total in P2 and P3.

The islet remodelling score (ESM Table 4) reflected disease progression from P1 to P3 with incrementally greater islet aggregation associated with increasing variability in size and shape. Peri-islet fibrosis was notable in P2 and P3 in addition to intra-islet fibrosis, with 5.8±4.7% (P2) and 8.6±6.8% (P3) of islets (on manual quantification) affected by significant fibrosis extending beyond the intra-islet perivascular region (ESM Figs. 13, 14).

Semi-quantitative scores for all features were significantly higher in CF compared with control blocks with the exception of inflammatory cell infiltration. ‘Mild inflammation’ was present in control donors and in CF without presence of ‘severe inflammation’ in any of the three histological patterns (Fig. 2f).

### AI analysis of pancreatic exocrine compartment

**Fig. 3:**
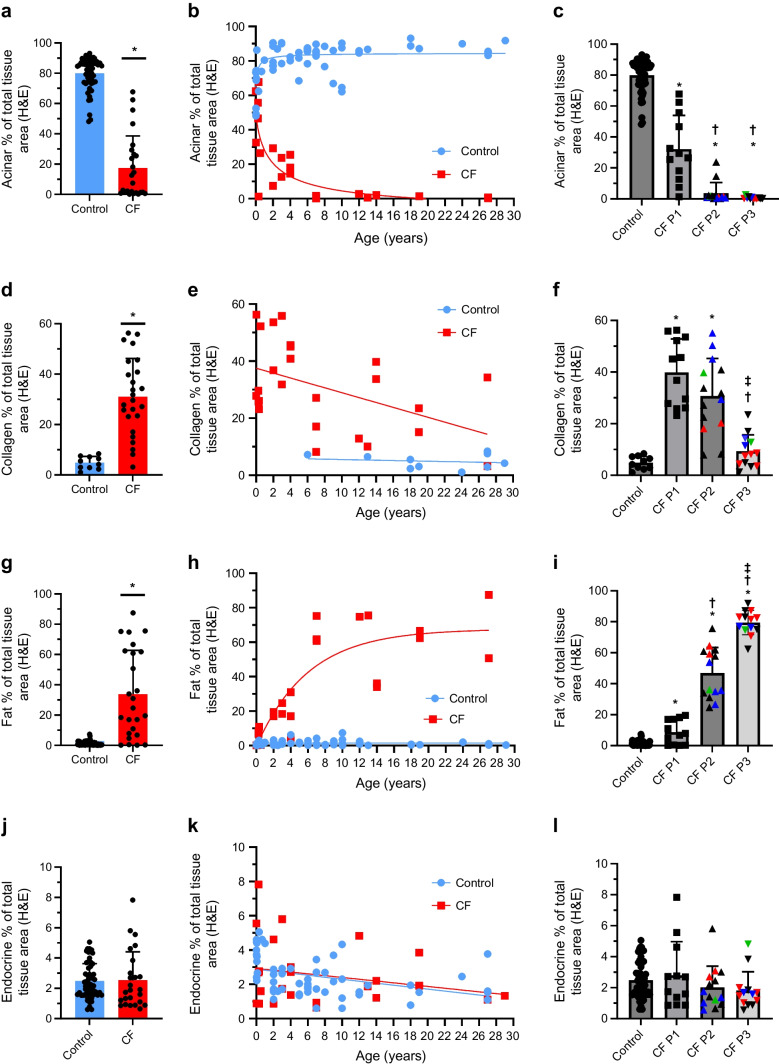
AI-based analysis of pancreatic histopathological features. The percentage of total pancreatic area is shown for the following areas: acinar (**a**–**c**); collagen (**d**–**f**); adipocyte (**g**–**i**); and endocrine (**j**–**l**). All control (blue) and CF (red) donors were compared by unpaired Student’s *t* test (**a**, **d**, **g**, **j**). Donor-based analysis vs age in control (blue circles) and CF (red squares) donors is shown in (**b**, **e**, **h**, **k**). For donors with multiple blocks, a mean value was calculated to obtain a single result per donor. Block-based analysis of control vs CF P1, P2 and P3 is shown in (**c**, **f**, **i**, **l**). A linear mixed effect model was used with histological CF pattern set as fixed effect, donor as random effect and Bonferroni post hoc test was used for statistical analysis. Graphs show mean ± SD. *p *< 0.05 was considered statistically significant. *p < 0.05 vs control; ^†^*p *< 0.05 vs CF P1; ^‡^*p *< 0.05 vs CF P2. In (**c**, **f**, **i**, **l**), red data points represent donor no. 22 and blue points represent donor no. 29 (both donors have multiple blocks of different patterns); green points represent CF donor no. 21 and CF donor no. 24 with diagnosed CFRD. *n *= 58 control donors, *n *= 25 CF donors (**a**, **b**, **g**, **h**, **j**, **k**). *n *= 10 control donors, *n *= 25 CF donors (**d**, **e**). *n *= 58 control blocks, *n *= 12 CF P1 blocks, *n *= 14 CF P2 blocks, *n *= 13 CF P3 blocks (**c**, **i**, **l**). *n *= 10 control blocks, *n *= 12 CF P1 blocks, *n *= 14 CF P2 blocks, *n *= 13 CF P3 blocks (**f**)

Quantification of stained sections from 25 CF and 58 age-matched control donors was undertaken. Approximately 80% of the pancreatic area comprised acinar tissue in control donors compared with mean area < 20% in donors with CF (Fig. 3a–c). In some very young CF donors, the acinar proportional area (PA) was similar to that of control donors but in others the acinar area was significantly reduced (Fig. 3b). No substantive acinar tissue remained by age 7 years in CF donors. This was reinforced in the block-level analysis showing significant reduction in acinar area in P1, with virtually no remaining acinar tissue in P2 or P3 (Fig. 3c).

Collagen area was significantly higher in CF donors, comprising >30% of overall area vs 5% in control donors (Fig. 3d–f). Collagen PA decreased with CF donor age in parallel with increasing (P2) and ultimately global (P3) adipocyte replacement. Overall collagen area in P3 pancreas was no longer significantly greater than in control pancreas.

Mean adipocyte area was 34% in CF donor pancreases compared with only 1% in control donors (Fig. 3g–i). The adipocyte areas in CF donors were greater than in control donors even in the youngest donors and increased rapidly with age and from P1 to P2 and ultimately P3. The percentage of adipose tissue in control pancreases remained very low regardless of age.

AI analysis for ductal area could not be meaningfully completed because the classifier could not accurately distinguish between blood vessels and pancreatic ducts.

### AI analysis of pancreatic endocrine compartment

The proportional endocrine area per section was comparable between control and CF cohorts and remained relatively stable with age in both groups (Fig. 3j–l). There were no statistically significant differences between the different CF patterns.

**Fig. 4:**
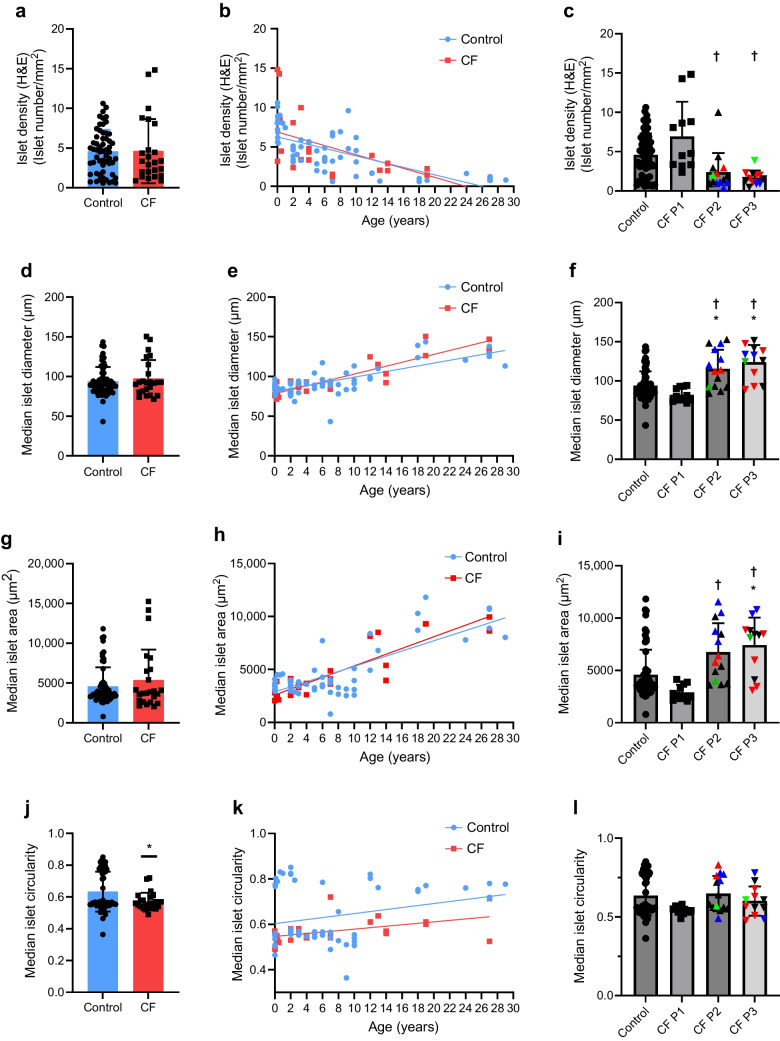
AI-based analysis of islet density (**a**–**c**), diameter (**d**–**f**), area (**g**–**i**) and circularity (**j**–**l**). All control (blue) and CF (red) donors were compared by unpaired Student’s *t* test (**a**, **d**, **g**, **j**). Donor-based analysis vs age in control (blue circles) and CF (red squares) donors is shown in (**b**, **e**, **h**, **k**). Block-based analysis of control vs CF P1, P2 and P3 is shown in (**c**, **f**, **i**, **l**). Linear mixed effect model was used with pancreatic pattern set as fixed effect, donor as random effect and Bonferroni post hoc test was used for statistical analysis. Graphs show mean of data points ± SD; data points are the median parameters calculated per section by the image analysis software. *p < 0.05 vs control; †*p *< 0.05 vs P1. In (**c**, **f**, **i**, **l**), red data points represent donor no. 22 and blue points represent donor no. 29 (both donors have multiple blocks of different patterns); green points represent CF donor no. 21 and CF donor no. 24 with diagnosed CFRD. *n *= 58 control donors, *n *= 24 CF donors (**a**, **b**, **d**, **e**, **g**, **h**, **j**, **k**). *n *= 58 control blocks, *n *= 11 CF P1 blocks, *n *= 14 CF P2 blocks, *n *= 12 CF P3 blocks (**c**, **f**, **i**, **l**)

Islet density was also comparable between control and CF pancreases overall (Fig. 4a–c). The density decreased markedly with increasing age in both groups. Islet density in sections with CF P2 and P3 was significantly lower than in P1.

Individual islet size, determined by diameter (Fig. 4d–f) and area (Fig. 4g–i), did not differ significantly between CF and control donors but increased linearly with donor age in both groups. In donors with CF, islets were significantly larger in P2 and P3 compared with P1 (Fig. 4f, i). Whether this increase in islet size over the first three decades of life in CF represents a true increase in endocrine mass per islet, mirroring normal growth, or an expansion of islet area by interposing fibrous tissue between endocrine cells in advanced CF remains unclear. The ability of AI to successfully differentiate individual islets within clusters was evidenced by highest median diameters of approximately 150 µm. Islet circularity was lower in CF donors than in control donors (Fig. 4j–l) without any significant changes with donor age or CF pattern.

### Changes in endocrine cell composition within CF pancreas

**Fig. 5:**
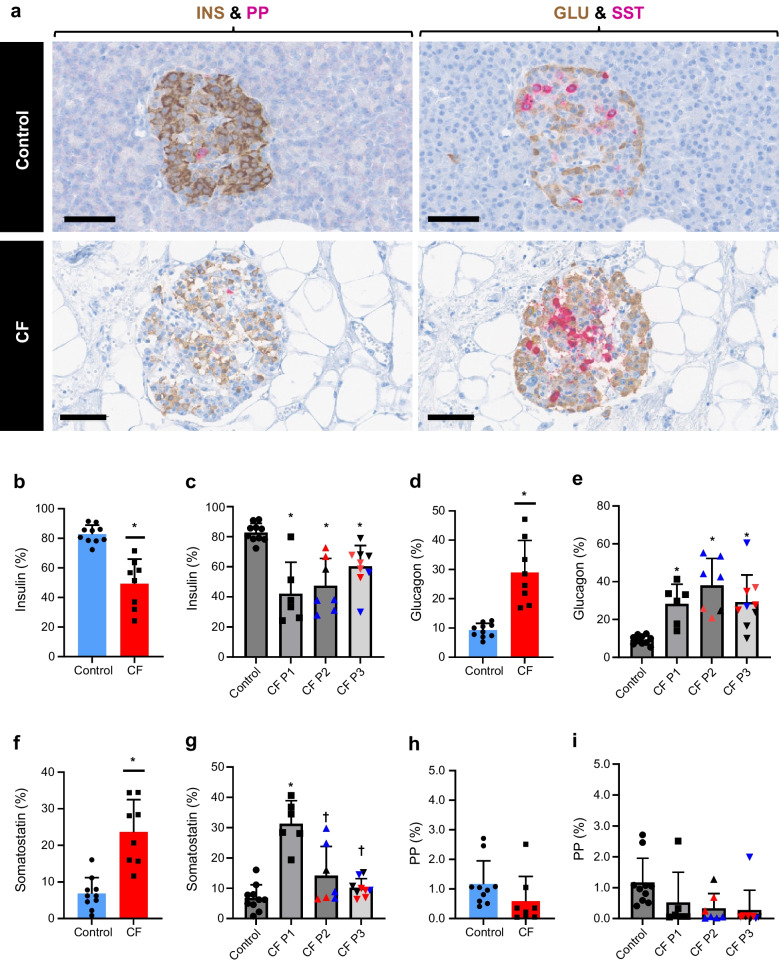
AI analysis of islet hormone composition in CF and control pancreases. (**a**) Dual immunohistochemistry staining for insulin (brown)/PP (pink) and glucagon (brown)/somatostatin (pink) in a control pancreas (donor no. 56) and CF pancreas (donor no. 29). Scale bars, 60 µm (control) or 70 µm (CF). (**b**–**i**) Donor-based AI analysis of insulin (**b**), glucagon (**d**), somatostatin (**f**) and PP (**h**) PA in control (blue) and CF (red) pancreases and block-based AI quantification of insulin (**c**), glucagon (**e**), somatostatin (**g**) and PP (**i**) PA are shown. The percentage area for each hormone was calculated as a proportion of the overall immunostained endocrine area (four stains combined). Donor-based comparison was carried out using unpaired Student’s *t* test and block-based comparisons were conducted using linear mixed effect model (LMEM) with histological CF pattern set as fixed effect, donor as random effect and Bonferroni post hoc test. Graphs show the mean ± SD. *p < 0.05 vs control; †*p *< 0.05 vs CF P1. In (**c**, **e**, **g**, **i**), red data points represent donor no. 22 and blue points represent donor no. 29 (both donors have multiple blocks with different CF patterns).* n *= 10 control donors, *n *= 8 CF donors (**b**, **d**, **f**, **h**). *n *= 10 control blocks, *n *= 6 CF P1 blocks, *n *= 7 CF P2 blocks, *n *= 9 CF P3 blocks (**c**, **e**, **g**, **i**). GLU, glucagon; INS, insulin; SST, somatostatin

Serial sections from the Klöppel CF cohort and the ten QUOD deceased organ donors were stained for insulin/PP and glucagon/somatostatin. Insulin PA was significantly lower in CF (49±17%) vs control donors (83±6%) (Fig. 5). In parallel, glucagon and somatostatin PAs were significantly increased in CF donors. PP area was comparable in CF and control donors.

Decreased beta cell and increased alpha cell PAs were evident from birth in CF, with differences from control donors remaining stable with age (ESM Fig. 15) and no evidence of further proportional beta cell loss with exocrine disease progression (Fig. 5c, e). Somatostatin PA was highest perinatally and decreased with age in donors with and without CF, being significantly higher in P1 than in control blocks but decreasing significantly in P2 and P3. No significant differences in PP PA were seen between any CF pattern and control blocks.

### Increased islet fibrosis in CF

**Fig. 6:**
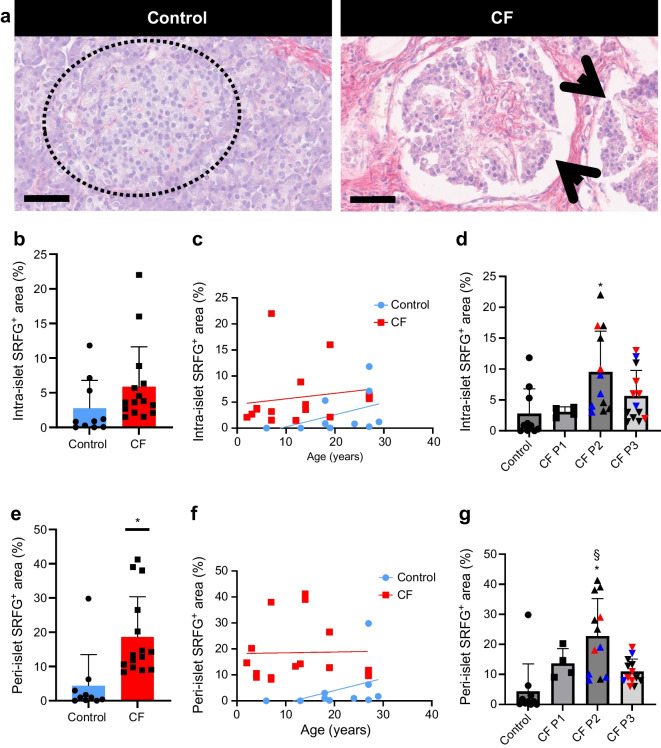
Collagen in the islet microenvironment in CF and control pancreases. (**a**) Representative images of SRFG staining of a control pancreas (donor no. 50) and CF pancreas (donor no. 22). Dashed circle indicates islet in control tissue, black arrows indicate islets in CF tissue. Scale bar, 60 µm. (**b**) Donor-based AI analysis of intra-islet proportion of collagen in control (blue) and CF (red) pancreases. (**c**) Donor-based analysis by age of control (blue) and CF (red) pancreases. (**d**) Block-based AI analysis of intra-islet proportion of collagen. (**e**) Donor-based AI analysis of peri-islet proportion of collagen in control (blue) and CF (red) pancreases. (**f**) Donor-based analysis by age of control (blue) and CF (red) pancreases. (**g**) Block-based AI analysis of peri-islet proportion of collagen. Donor-based statistical analyses were carried out using unpaired Student’s *t* test and block-based analyses were conducted using linear mixed effect model with CF histological pattern set as fixed effect, donor as random effect and Bonferroni post hoc test. Graphs show the mean ± SD. *p < 0.05 vs control; §*p *< 0.05 vs CF P3. In (**d**, **g**), red data points represent donor no. 22 and blue points represent donor no. 29 (both donors have multiple blocks with different CF patterns). *n *= 10 control donors, *n *= 15 CF donors (**b**, **c**, **e**, **f**). *n *= 10 control blocks, *n *= 4 CF P1 blocks, *n *= 12 CF P2 blocks, *n *= 13 CF P3 blocks (**d**, **g**)

SRFG PA within islets was quantified in the Klöppel CF cohort in comparison with the ten QUOD organ donors. Intra-islet fibrosis was threefold higher in CF donors (Fig. 6). In contrast to decreasing overall pancreatic fibrosis with age and progressive pathology towards adipocyte replacement (Fig. 3), islet fibrosis persisted in the oldest CF donors and in the CF donors with P3 (Fig. 6).

### Activated stellate cell and leucocyte distribution

**Fig. 7:**
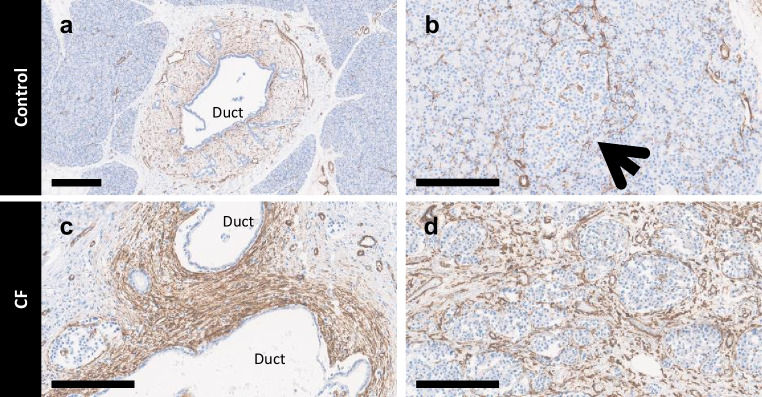
Distribution of stellate cells in CF and control pancreases. Immunostaining for α-SMA (brown) in control pancreases (**a** [donor no. 31]; **b** [donor no. 51]) and CF pancreases (**c** [donor no. 22]; **d** [donor no. 27]) indicating PSCs. (**a**, **c**) α-SMA staining in the periductal stroma. (**b**, **d**) α-SMA staining in the islet microenvironment. Black arrow indicates islet. Scale bar, 300 µm (**a**) or 200 µm (**b**–**d**)

Qualitative analysis of α-SMA staining pattern as a marker of activated pancreatic stellate cells (PSCs) [20] was undertaken in 31 CF donor blocks and sections from the QUOD control donor cohort [20] (Fig. 7). While some positive staining was seen in control donors, relatively high numbers and densities of α-SMA-expressing cells were seen in areas of fibrosis surrounding ducts and islets in donors with CF (Fig. 7). Cellular morphology and topography in addition to CD31 immunostaining was used to differentiate stellate from endothelial cells and other perivascular cells.

Through CD45 staining of Klöppel CF cohort blocks and slides from QUOD control donors, leucocytes could be identified in all slides examined (with the exception of a single donor with CF [donor no. 3]). Numbers were higher around vascular structures, with an example in a control donor shown in ESM Fig. 16. In many donors with CF, foci of lymphocytic inflammation were observed (ESM Fig. 16). In some donors with CF, there was evidence of increased leucocyte density around ducts and islets (ESM Fig. 16).

CF pancreatic blocks stained for CD68 showed macrophages scattered in the stroma, within inflammatory cell foci and with a peri- and intra-islet distribution in some sections (ESM Fig. 17).

AI-assisted quantification of α-SMA and CD45 PA was challenging in post mortem CF donors due to potential under-staining in comparison with deceased organ donors, disrupted tissue morphology and loss of cellularity with disease progression (ESM Figs 18, 19). Overall leucocyte numbers were significantly lower in CF donors in keeping with artefactual under-staining but counts did not increase with CF age or disease stage (ESM Fig. 18). Loss of α-SMA PA was seen with increasing age in CF and progression to P3 global adipocyte replacement (ESM Fig. 18). Peri- and intra-islet α-SMA-expressing cells persisted even in end-stage disease (ESM Fig. 19).

## Discussion

CF pancreatic pathology has been described qualitatively [14, 17, 21–25] with two patterns of exocrine pancreatic pathology, ‘fibrotic’ and ‘lipoatrophic’, well-recognised in adults, with an association between CFRD and extensive fat replacement [16, 17, 26]. Through qualitative evaluation of a CF cohort with a wide age range it became clear that each CF tissue block could be classified into one of three (rather than two) patterns. Through systematic semi-quantitative and AI-enhanced quantitative analysis we compared CF with age-matched control donors, plotted data according to donor age and undertook comparative analysis according to these three patterns, enabling us to define a clear natural history.

Early stage ‘fibrosis’ alone was characterised by mild duct dilatation with bands of interlobular fibrosis. Lobularity of exocrine parenchyma was largely preserved, with focal acinar cell loss and replacement by fibrosis. In advanced stage ‘fibrosis/liposis’, (virtually) all acinar tissue was lost, with growing areas of parenchymal adipocyte replacement in addition to extensive confluent fibrosis surrounding distinctly dilated ducts and focal groups of islets surrounded by liposis or fibrosis. End-stage ‘lipotic’ organs were atrophic, with the majority of the pancreas comprising mature adipose tissue but with maintained mass of densely clustered islets surrounded and traversed by collagen strands and with any identifiable residual ducts also surrounded by fibrosis.

The rapid increase in AI-quantified adipocyte area with increasing age culminating in virtual complete replacement of the exocrine pancreas provides convincing evidence for inexorably progressive fat replacement in the CF pancreas during prolonged complete duct blockage with progressively severe periductal fibrosis. While aetiological drivers of fat replacement in CF remain unknown, we have detected upregulation of genes associated with fatty acid metabolism and adipogenesis in Nanostring analysis of P2/P3 pancreases (Y. Al-Selwi , N. Kattner, J. A. M. Shaw, unpublished data). Upregulation of adipogenic genes within the pancreas has also been described in a CF ferret model [27].

Our pathological data are in keeping with imaging studies demonstrating replacement of the gland almost entirely by fat in all adults with CF and PEI [24, 25]. In a pilot study (B. Nielsen, D. Faurholt-Jepsen, unpublished data) in nine people with CF aged >18 years, pancreatic fat percentage measured by MRI was >75% regardless of glucose tolerance from normal to CFRD. In a separate study an association between pancreatic fat replacement and degree of exocrine but not endocrine dysfunction was shown [25]. Moreover, Shwachman’s syndrome, a hereditary disorder associated with replacement of pancreatic parenchyma by adipocytes without fibrosis, is associated with PEI but not diabetes [17, 28]. We conclude that adipocyte replacement is unlikely to be the primary driver of CFRD.

Our analysis provides evidence for changes in the CF pancreas at, and likely preceding, birth. In line with the findings of Bogdani et al [16], dilated ducts and widening of the interlobular areas with bands of collagen fibres were seen in infants with CF. These findings suggest initiation of the remodelling process within the ductal compartment supported by ductal localisation of CF transmembrane conductance regulator (CFTR) expression in human pancreas [13, 29, 30].

A relative decrease in acinar volume has been reported even at birth [31], with increasing acinar loss with age in very young CF donors [16]. We quantified the rate of acinar cell loss over a wide age range and demonstrated near-complete acinar atrophy by the age of 7 years. This is confirmed clinically by early onset PEI [1, 6, 32–34].

Pancreatic fibrosis is a central finding [16, 17, 21, 26, 35]. Our study included evaluation of spatial distribution, initially localised to the periductal area, with severity mirroring the degree of ductal dilatation. Exocrine fibrosis was greatest in CF P1 and decreased in older donors as previously described [17, 26], with involution paralleling ductal loss. Residual small ducts in end-stage lipotic CF were surrounded by persisting fibrosis.

Intra-islet fibrosis has been observed previously but has not been quantified [15–17, 21, 22, 26]. While overall pancreatic fibrosis decreased with progression from CF P1 to P3, intra-islet fibrosis peaked later (P2) and persisted in the lipotic gland, with collagen making up a significant proportion of overall islet area in P3.

We confirmed a cellular component to the initial periductal and later peri-islet fibrosis in CF through presence of α-SMA-positive activated PSCs. In addition, inflammatory cell foci comprising predominantly lymphocytes/histiocytes and periductal/peri-islet leucocytes including macrophages were seen. Increased numbers of immune cells including macrophages around and within islets have been reported [15, 16, 36].

Activated PSCs in association with macrophages are believed to play a critical role in fibrogenesis and tissue remodelling in chronic pancreatitis [20, 37–42]. Periductal activated PSCs have been identified in the CF ferret but not in wild-type controls and it has been hypothesised that these are key to pancreatic exocrine destruction and adipogenesis [43].

AI quantification demonstrated no significant differences between the per cent endocrine area, islet density or size in CF pancreases from premature birth to 27 years of age compared with age-matched controls. Decreasing islet density with age was mirrored by increasing area of individual islets in both cohorts and the similarity between CF and control donors with increasing age over the first 30 years of life was striking. There was no significant change in percentage of the overall pancreatic area comprising islets with increasing age in CF or control donors. Our systematic well-controlled unbiased AI analysis adds to previously reported findings that CF is not associated with significant reduction in overall islet mass [16, 17, 23]. A possible decrease in endocrine mass has been reported [16, 26] by some groups but we conclude that although some loss in advanced CF cannot be ruled out, this is at worst modest.

Islet circularity was lower in CF and semi-quantitative scoring revealed progressive remodelling of the pancreatic endocrine compartment with islet aggregation within complexes surrounded by fibrosis and activated PSCs in P2 and islets with variable size and shape including a proportion with extensive intra-islet fibrosis in P3. Even in the presence of global fat replacement and gland atrophy, all islet clusters were associated with surrounding collagen strands and residual fibrosis [16, 17, 21, 22, 26]. We hypothesise that peri- and intra-islet fibrosis play a key role in pancreatic endocrine dysfunction and ultimately in progression to diabetes and that this process may be mediated by activated PSCs. We propose that activated PSCs orchestrate reorganisation of the islet niche, disrupting endocrine cell-to-cell communication critical for normal function and potentially also normal blood flow through vascular ‘strangulation’ [17]. In addition to fibrogenesis, the PSC secretome may directly impair proliferation, viability and function of endocrine cells [41, 44–46].

It appears that pancreatic fibrosis in the earlier stages of CF may be dependent on the presence of ‘stressed’ dilated ducts leading to PSC activation and collagen secretion, resolving as ducts are lost, with a second phase of fibrosis fuelled by the combination of ‘stressed islets’, macrophage and stellate cell activation leading to peri- and intra-islet fibrosis, islet dysfunction and ultimately diabetes.

Within islets, insulin PA was reduced by almost 50% in CF P1 donors in comparison with control donors. This was the case even in donors dying soon after birth, in agreement with a previous study investigating very young CF donors [16]. Reduced beta cell PA remained relatively constant with CF donor age and CF pattern (P2/3). The absence of further beta cell loss during development of abnormal blood glucose levels and even after progression to CFRD is reflected in all other published literature relating to human donors [15–17, 21–23]. Decreased insulin area was mirrored by a threefold increase in glucagon PA and this again remained relatively stable with age and exocrine pancreatic disease progression in keeping with the majority of previous publications [15–17, 36]. Somatostatin PA was threefold higher in P1 CF donors compared with non-CF control donors as previously reported [16, 17, 36]. This decreased not only with CF disease progression but also with increasing age in donors without CF, potentially reflecting normal postnatal development rather than CF-specific pathology.

Strengths of our study include the relatively large number of CF donors obtained by pooling several tissue repositories and applying a systematic approach including semi-quantitative analysis in parallel with unbiased AI image analysis. This approach has potential for application beyond CF for robust scoring of both exocrine and endocrine compartments in other pancreatic diseases including chronic pancreatitis [47]. Weaknesses include autolytic changes within post mortem tissue affecting staining quality. Further, gross disruption of pancreatic parenchyma in advanced CF impacted the accuracy of AI classification. Limited donor data were available for the historic CF donors; ante mortem diabetes status and CF genotype were unknown, precluding any firm conclusions regarding impact of the pathological patterns observed on islet dysfunction and diabetes. Absence of data on donor sex in the EADB cohort precluded any assessment of potential differences between male and female donors.

In summary, our analysis has demonstrated a biphasic pattern of pancreatic fibrosis in CF: duct blockage initiates a predominantly periductal fibrosis during development of PEI; followed by peri-islet fibrosis potentially driven by activated stellate cells and macrophages and associated with islet clustering and progressive disorganisation. This process culminates in extensive intra-islet collagen deposition potentially interrupting normal endocrine cell-to-cell communication and restricting islet blood flow.

It now appears clear that CFTR modulators commenced in individuals aged >12 years, and therefore with ‘lipotic’ pancreases, cannot prevent progression to diabetes despite their transformative impact on CF-related pulmonary disease [48]. Identification of a cellular fibrotic process in and around an islet compartment with maintained beta cell mass in later-stage pancreatic CF suggests the potential for novel anti-fibrotic therapeutic approaches to restore beta cell function and alleviate the secondary diabetes-associated morbidity increasingly associated with CFRD in adulthood [49].

## Supplementary Information

Below is the link to the electronic supplementary material.

ESM (PDF 3825 KB)

## Data Availability

Data are available from the corresponding author upon reasonable request.

## Abbreviations

AI: Artificial intelligence
CF: Cystic fibrosis
CFRD: CF-related diabetes
CFTR: CF transmembrane conductance regulator
CGA: Chromogranin A
EADB: Exeter Archival Diabetes Biobank
P1, P2, P3: Pattern 1, Pattern 2, Pattern 3
PA: Proportional area
PEI: Pancreatic exocrine insufficiency
PP: Pancreatic polypeptide
PSC: Pancreatic stellate cells
QUOD: Quality in Organ Donation
QUOD-PANC: Quality in Organ Donation Whole Pancreas Biobank
SMA: Smooth muscle actin
SRFG: Sirius Red Fast Green
