# Supplementary material for: Generation of a pseudo-timeline describing progressive human exocrine and endocrine pancreatic pathology in cystic fibrosis through novel semi-quantitative scoring and AI-driven quantitative image analysis
Source: Diabetologia. 2025 Oct 12;69(1):157–72. doi: 10.1007/s00125-025-06559-4 (PMC12686001; doi:10.1007/s00125-025-06559-4)
Supplement: Supplementary file 1 — ESM (PDF 3825 KB) [file 125_2025_6559_MOESM1_ESM.pdf]

ESM Table 1: CF donors

Case Number	Biobank	Age	Sex	Number of blocks	CF Pattern	Stain
1	Klöppel	Premature	Female	1	Pattern 1	H&E, SRFG, CGA / CD31, INS / PP, GLU / SST, alpha-SMA, CD45
2	Klöppel	Premature	Female	3	Pattern 1	H&E, SRFG, CGA / CD31, INS / PP, GLU / SST, alpha-SMA, CD45, CD68 (2 / 3 blocks)
3	Klöppel	2 days	Male	1	Pattern 1	H&E, SRFG, CGA / CD31, INS / PP, GLU / SST, alpha-SMA, CD45
4	EADB	3 days	NA	1	Pattern 1	H&E, SRFG, CGA / CD31
5	EADB	10 days	NA	1	Pattern 1	H&E, SRFG, CGA / CD31
6	EADB	3 months	NA	1	Pattern 1	H&E, SRFG, CGA / CD31
7	EADB	3 months	NA	1	Pattern 1	H&E, SRFG, CGA / CD31
8	EADB	4 months	NA	1	Pattern 1	H&E, SRFG, CGA / CD31
9	Klöppel	4 months	Male	2	Pattern 1	H&E, SRFG, CGA / CD31, INS / PP, GLU / SST, alpha-SMA, CD45, CD68 (1 / 2 blocks)
10	EADB	6 months	NA	1	Pattern 1	H&E, SRFG, CGA / CD31
11	EADB	2 years	NA	1	Pattern 1	H&E, SRFG, CGA / CD31
12	EADB	2 years	NA	1	Pattern 1	H&E, SRFG, CGA / CD31
13	EADB	3 years	NA	1	Pattern 1	H&E, SRFG, CGA / CD31
14	EADB	3 years	NA	1	Pattern 2	H&E, SRFG, CGA / CD31
15	EADB	4 years	NA	1	Pattern 1	H&E, SRFG, CGA / CD31
16	EADB	4 years	NA	1	Pattern 1	H&E, SRFG, CGA / CD31
17	EADB	4 years	NA	1	Pattern 2	H&E, SRFG, CGA / CD31
18	EADB	7 years	NA	1	Pattern 2	H&E, SRFG, CGA / CD31
19	EADB	7 years	NA	1	Pattern 3	H&E, SRFG, CGA / CD31

20	Klöppel	7 years	Male	1	Pattern 2	H&E, SRFG, CGA / CD31, INS / PP, GLU / SST, alpha-SMA, CD45
21*	EADB	12 years	NA	1	Pattern 3	H&E, SRFG, CGA / CD31
22	Klöppel	13 years	Female	7	Pattern 3 (5 blocks) Pattern 2 (2 blocks)	H&E, SRFG, CGA / CD31, INS / PP, GLU / SST, alpha-SMA, CD45, CD68 (6 / 7 blocks)
23	EADB	14 years	NA	1	Pattern 2	H&E, SRFG, CGA / CD31
24*	EADB	14 years	NA	1	Pattern 2	H&E, SRFG, CGA / CD31
25	Klöppel	14 years	Female	1	Pattern 2	H&E, SRFG, CGA / CD31, INS / PP, GLU / SST, alpha-SMA, CD45
26	EADB	19 years	NA	1	Pattern 3	H&E, SRFG, CGA / CD31
27	Klöppel	19 years	Male	2	Pattern 2	H&E, SRFG, CGA / CD31, INS / PP, GLU / SST, alpha-SMA, CD45, CD68 (1 / 2 blocks)
28	Klöppel	27 years	Male	3	Pattern 3	H&E, SRFG, CGA / CD31, INS / PP, GLU / SST, alpha-SMA, CD45, CD68 (2 / 3 blocks)
29	Klöppel	27 years	Male	6	Pattern 2 (4 blocks) Pattern 3 (2 blocks)	H&E, SRFG, CGA / CD31, INS / PP, GLU / SST, alpha-SMA, CD45, CD68 (5 / 6 blocks)
		Mean age: 6.31 years				

Pattern 1= Fibrotic, Pattern 2= Fibrotic and lipotic, Pattern 3= Lipoatrophic. Not available (NA). EADB cohort: Exeter Archival Diabetes Biobank. Klöppel cohort: Provided by Prof. Günter Klöppel. Donors marked with an asterisk have a confirmed diabetes diagnosis. Cause of death was meconium ileus in Cases 1-5; failure to thrive in Cases 6-10; respiratory infection in Cases 11-29.

ESM Table 2: Control donors

Case No.	Biobank	Age	Sex	Stain
1	EADB	0	NA	H&E
2	EADB	7 days	NA	H&E
3	EADB	7 days	NA	H&E
4	EADB	7 days	NA	H&E
5	EADB	7 days	NA	H&E
6	EADB	13 days	NA	H&E
7	EADB	3 weeks	NA	H&E
8	EADB	3 weeks	NA	H&E
9	EADB	3 weeks	NA	H&E
10	EADB	3 weeks	NA	H&E
11	EADB	6.2 weeks	NA	H&E
12	EADB	4 months	NA	H&E
13	EADB	8 months	NA	H&E
14	EADB	1 year	NA	H&E
15	EADB	2 years	NA	H&E
16	EADB	2 years	NA	H&E
17	EADB	2 years	NA	H&E
18	EADB	2 years	NA	H&E
19	EADB	2 years	NA	H&E
20	EADB	2 years	NA	H&E
21	EADB	2 years	NA	H&E
22	EADB	2.5 years	NA	H&E
23	EADB	3 years	NA	H&E
24	EADB	3 years	NA	H&E
25	EADB	3 years	NA	H&E
26	EADB	4 years	NA	H&E
27	EADB	4 years	NA	H&E
28	EADB	5 years	NA	H&E
29	EADB	5 years	NA	H&E
30	EADB	5 years	NA	H&E

31	QUOD	6 years	Female	H&E, SRFG, CGA / CD31, CD45, INS / PP, GLU / SST, alpha-SMA
32	EADB	6 years	NA	H&E
33	EADB	6 years	NA	H&E
34	EADB	6 years	NA	H&E
35	EADB	6 years	NA	H&E
36	EADB	7 years	NA	H&E
37	EADB	7 years	NA	H&E
38	EADB	7 years	NA	H&E
39	EADB	7 years	NA	H&E
40	EADB	8 years	NA	H&E
41	EADB	8 years	NA	H&E
42	EADB	9 years	NA	H&E
43	EADB	9 years	NA	H&E
44	EADB	10 years	NA	H&E
45	EADB	10 years	NA	H&E
46	EADB	10 years	NA	H&E
47	EADB	10 years	NA	H&E
48	EADB	12 years	NA	H&E
49	EADB	12 years	NA	H&E
50	QUOD	13 years	Male	H&E, SRFG, CGA / CD31, CD45, INS / PP, GLU / SST, alpha-SMA
51	QUOD	18 years	Female	H&E, SRFG, CGA / CD31, CD45, INS / PP, GLU / SST, alpha-SMA
52	QUOD	18 years	Female	H&E, SRFG, CGA / CD31, CD45, INS / PP, GLU / SST, alpha-SMA
53	QUOD	19 years	Male	H&E, SRFG, CGA / CD31, CD45, INS / PP, GLU / SST, alpha-SMA
54	QUOD	24 years	Male	H&E, SRFG, CGA / CD31, CD45, INS / PP, GLU / SST, alpha-SMA
55	QUOD	27 years	Male	H&E, SRFG, CGA / CD31, CD45, INS / PP, GLU / SST, alpha-SMA

56	QUOD	27 years	Male	H&E, SRFG, CGA / CD31, CD45, INS / PP, GLU / SST, alpha-SMA
57	QUOD	27 years	Male	H&E, SRFG, CGA / CD31, CD45, INS / PP, GLU / SST, alpha-SMA
58	QUOD	29 years	Male	H&E, SRFG, CGA / CD31, CD45, INS / PP, GLU / SST, alpha-SMA
		Mean age: 7.05 years		

EADB cohort: Exeter Archival Diabetes Biobank. QUOD: Quality in Organ Donation. NA: not available.

ESM Table 3: Antibody specifications

**Antibody**	**Reference**	**Dilution**	**Company**	**RRID**
Insulin	BSH-2010-100	1:1000	Nordic Biosite, Täby, Sweden	NA
Glucagon	EP74	1:1500	Cell Marque, California, USA	NA
Pancreatic polypeptide	ab113694	1:2000	Abcam, Cambridge, UK	AB_11156699
Somatostatin	EP130	1:300	Cell Marque, California, USA	NA
Chromogranin A	ab254557	1:5000	Abcam, Cambridge, UK	NA
CD45	2B11+PD7/26	1:250	Dako, Agilent Technologies, Santa Clara, California, USA	NA
CD31	ab182981	1:2000	Abcam, Cambridge, UK	AB_2920881
Alpha-SMA	ab150301	1:200	Abcam, Cambridge, UK	NA
CD68	M0876	1:200	Dako, Agilent Technologies, Santa Clara, USA	NA

Tissue was pre-treated with Discovery CC1 solution (Ventana Medical Systems, Roche Diagnostics Limited, UK) which is a EDTA based solution used for Ventana automated slide strainers. NA: Not available.

ESM Table 4: Semi-quantitative scoring parameters

Score	0	1	2	3
**Ductal lumen dilation**	Non-dilated	Little dilatation sometimes filled with mucus	Dilated and often filled with mucus	Massive dilatation often filled with mucus
**Ductal loss**	No ductal loss	Focal ductal loss	Moderate ductal loss	Severe ductal loss
**Exocrine pancreas fibrosis**	No abnormal presence of fibrotic tissue	Mild presence of fibrotic tissue	Moderate presence of fibrotic tissue	Massive presence of fibrotic tissue
**Acinar atrophy**	No acinar atrophy	<1/3 acini lost	1/3 – 2/3 acini loss	>2-3 acini loss
**Islet remodelling**	No abnormal size or distribution	Some solitary islets surrounded by fibrosis	Many islets are aggregated in complexes	Most islets of variable size and shape are aggregated in complexes
**Inflammation**	Scattered inflammatory cells	Mild inflammation	Moderate inflammation	Severe inflammation

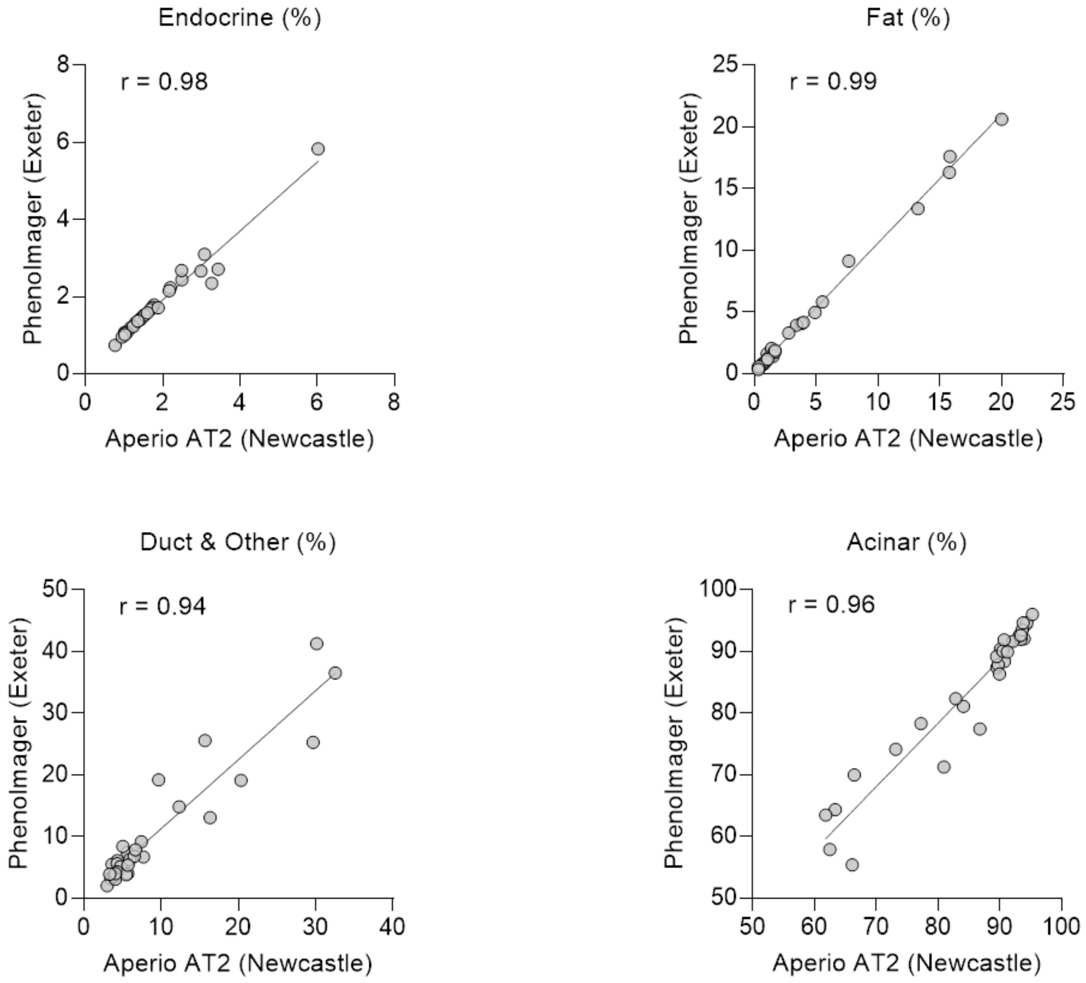

ESM Fig. 1: Comparison of percentage area measurements in 29 donors scanned with both scanners highlighting the correlation between both. This analysis included donors from the QUOD PANC biobank which were not part of the main study cohort.

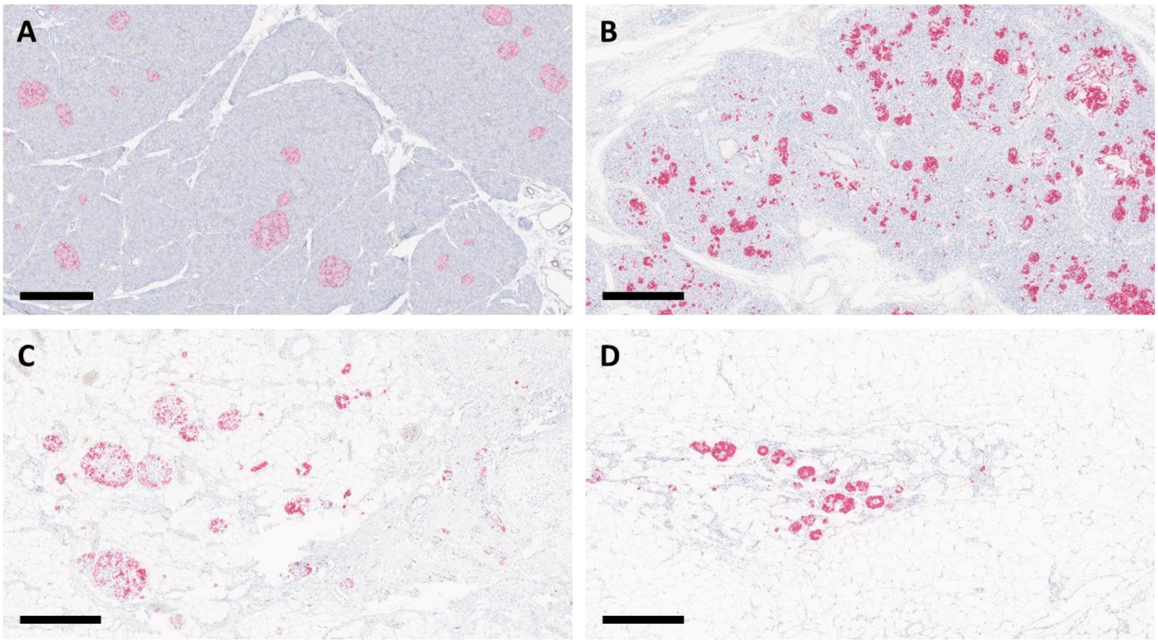

ESM Fig. 2: Representative images for control and CF patterns with CGA (pink) and CD31 (brown) staining. (A) Control pancreas (Control Case 53) with normal tissue and islet morphology. (B) CF Pattern 1 (CF Case 9). (C) CF Pattern 2 (CF Case 29). (D) CD Pattern 3 (CF Case 22). Scale bars: 500 µm.

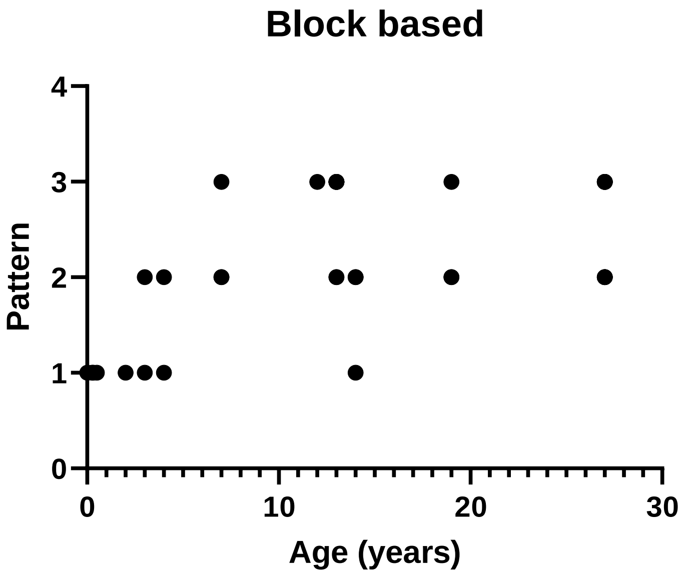

ESM Fig. 3: Block based pattern analysis of donors with CF vs age. Spearman correlation coefficient r=0.7156, p<0.0001.

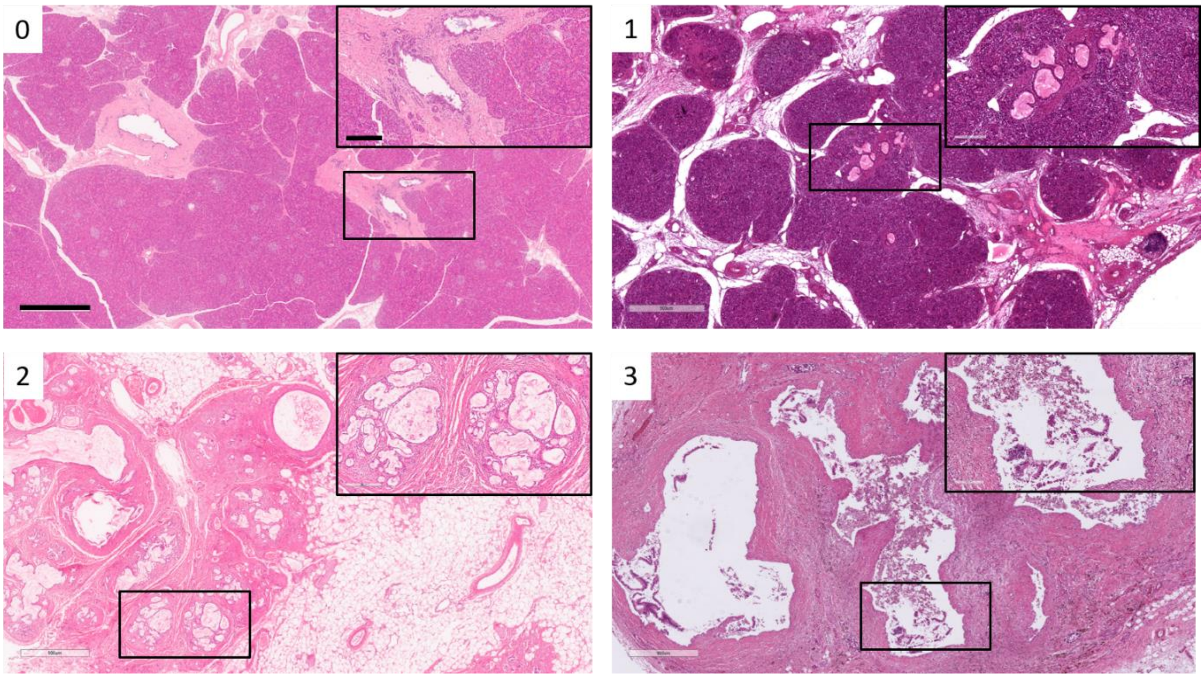

ESM Fig. 4: Representative H&E stained control (0) and CF (1-3) pancreatic tissue to exemplify the semi-quantitative scoring system for ductal lumen dilation. 0: non-dilated; 1: little dilatation sometimes filled with mucus; 2: ducts dilated and often filled with mucus; 3: massive dilatation. Scale bars 900 µm and 200 µm for magnified images (top right corner).

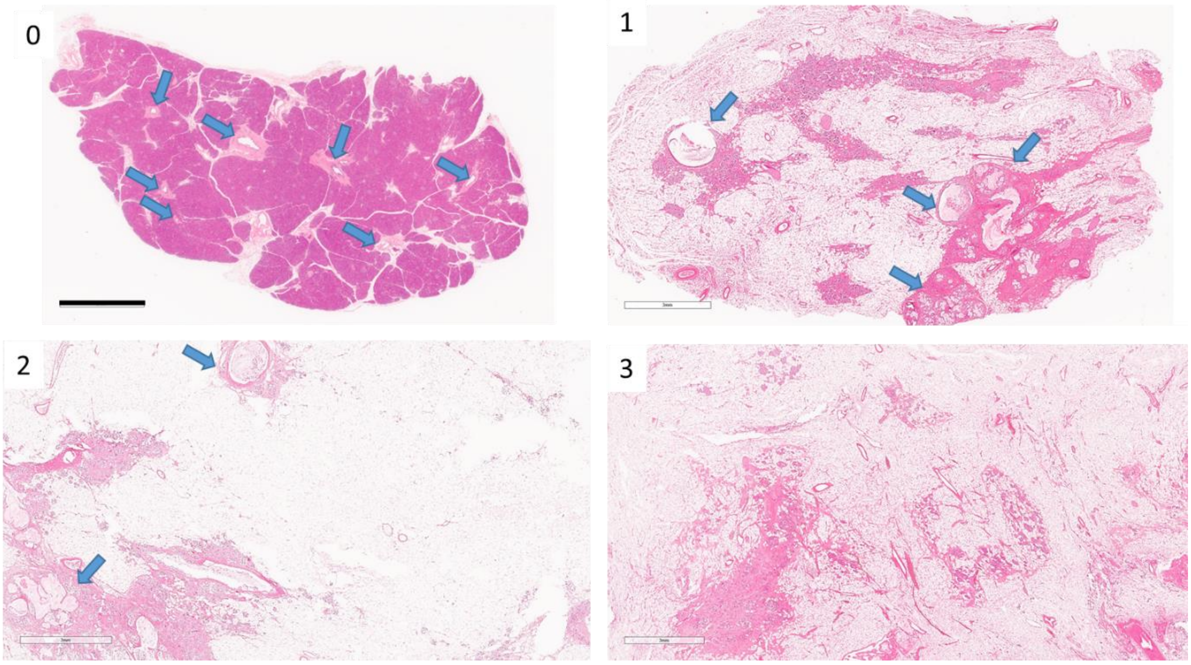

ESM Fig. 5: Representative H&E stained control (0) and CF pancreatic tissue (1-3) to exemplify the semi-quantitative scoring system for ductal loss. 0: no ductal loss; 1: focal loss; 2: moderate loss; 3: severe loss. Blue arrows indicate ducts. Scale bar 3 mm.

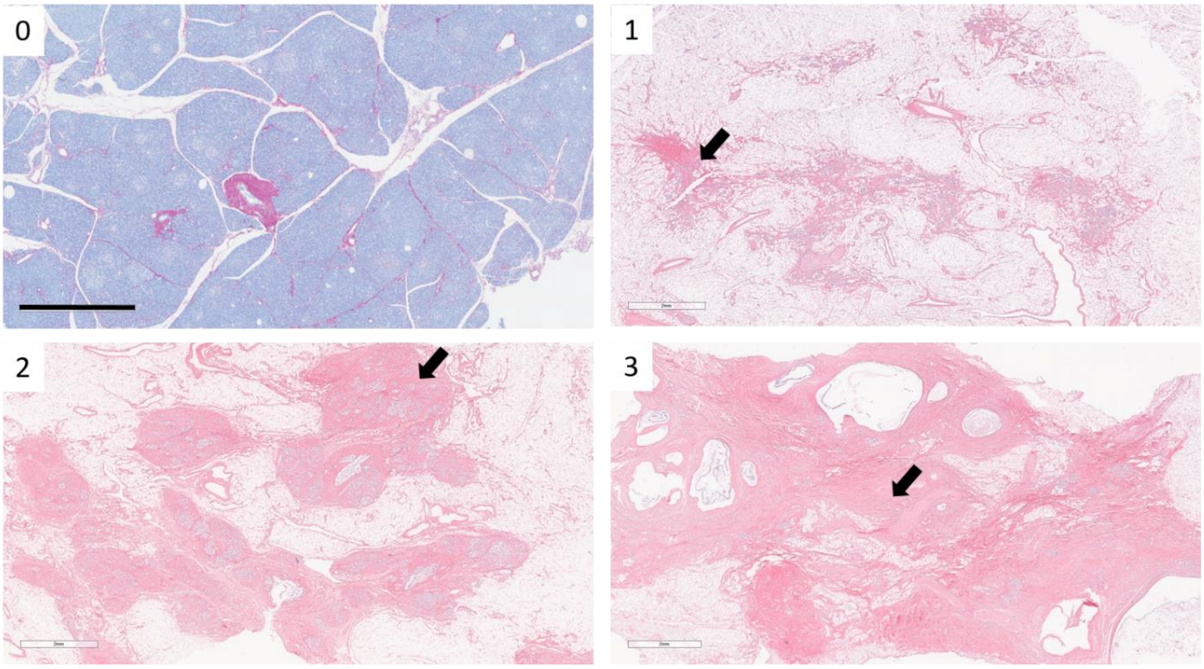

ESM Fig. 6: Representative SRFG stained control (0) and CF pancreatic tissue (1-3) to exemplify the semi-quantitative scoring system for exocrine pancreas fibrosis. 0: no abnormal presence of fibrotic tissue; 1: mild presence of fibrotic tissue; 2: moderate presence of fibrotic tissue; 3: massive presence of fibrotic tissue. Black arrows indicate examples of fibrotic areas. Scale bar 2 mm.

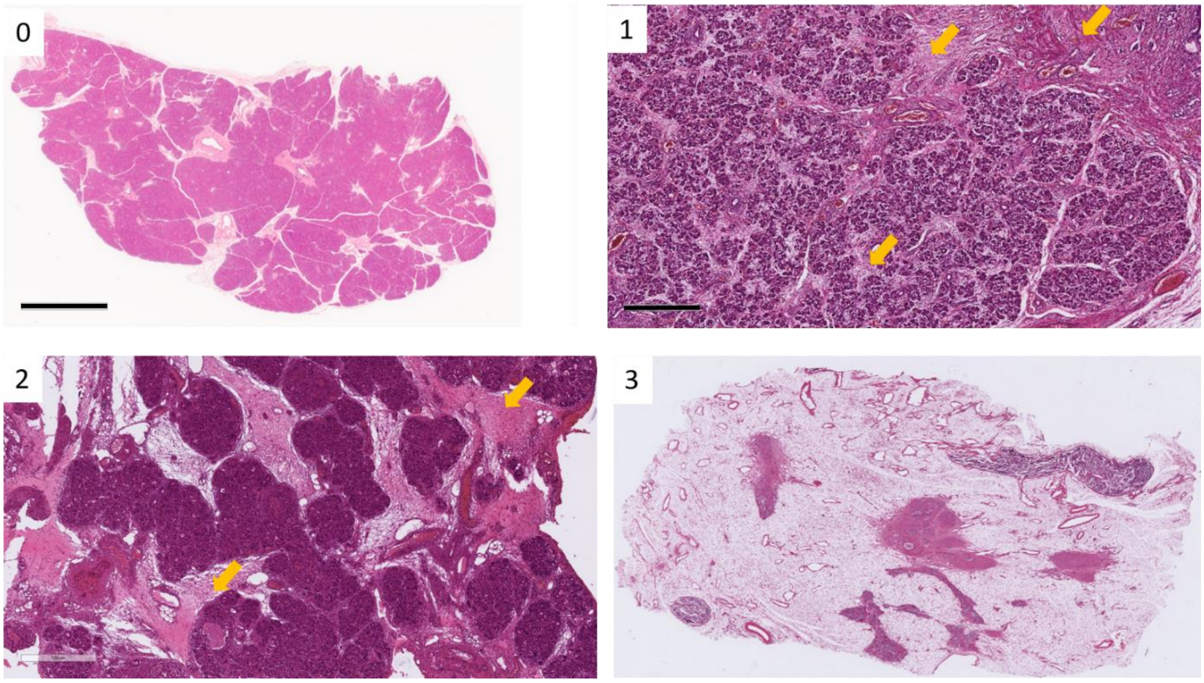

ESM Fig. 7: Representative H&E stained control (0) and CF pancreatic tissue (1-3) to exemplify the semi-quantitative scoring system for acinar atrophy. 0: no acinar atrophy; 1: < 1/3 of acini lost; 2: 1/3 – 2/3 acini loss; 3: >2/3 acini loss. Yellow arrows indicate example areas of acinar atrophy. Scale bar 500 µm (1 &2) and 4 mm (0&3).

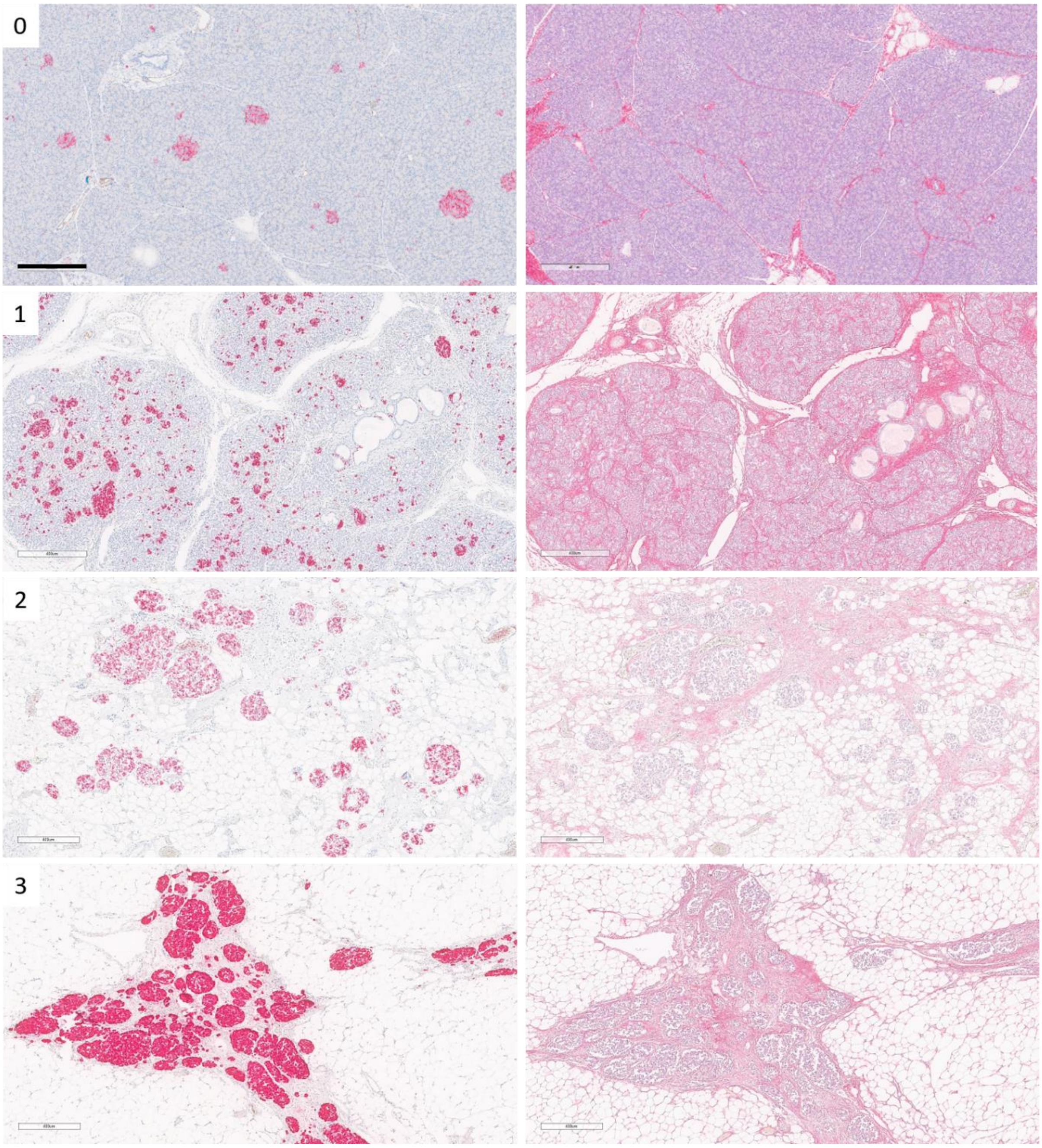

ESM Fig. 8: Representative Chromogranin A (left column) and SRFG (right column) stained control (0) and CF pancreatic tissue (1-3) to exemplify the semi-quantitative scoring system for islet remodelling. 0: no abnormal size or distribution; 1: some solitary islets surrounded by fibrosis; 2: many islets are aggregated in complexes; 3: most islets of variable size and shape are aggregated in complexes. Scale bar 400 µm.

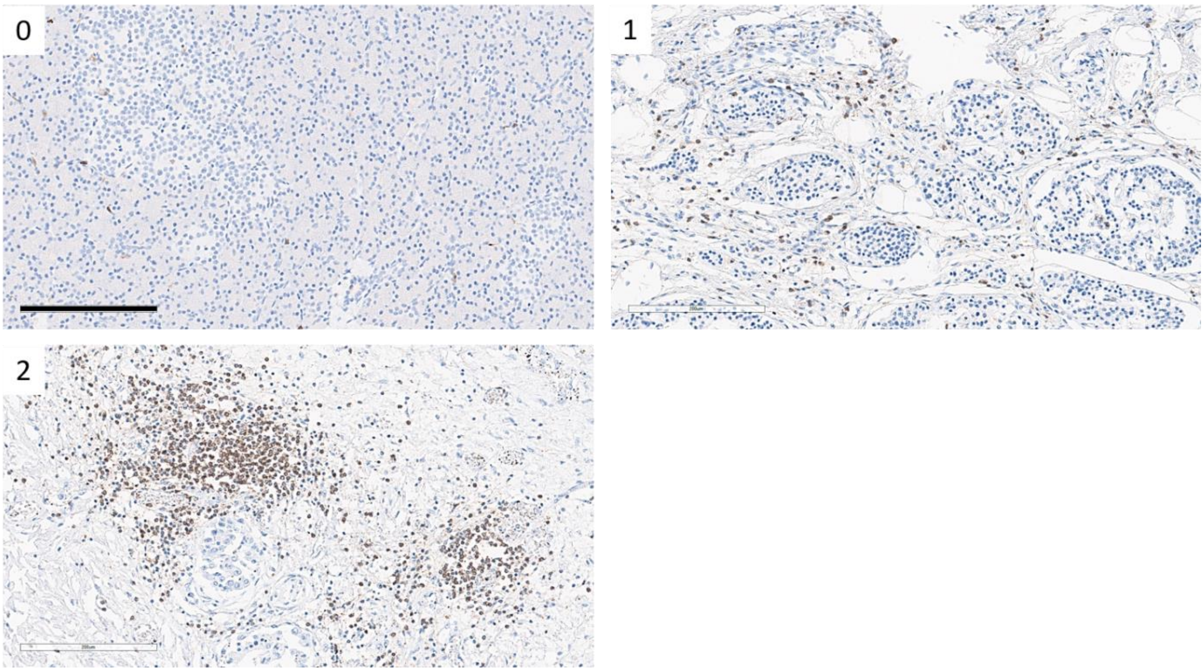

ESM Fig. 9: **Representative CD45 (brown) stained control (0) and CF pancreatic tissue (1-2) to exemplify the semi-quantitative scoring system for inflammation.** 0: scattered inflammatory cells; 1: mild inflammation; 2: moderate inflammation. Scale bar 200 µm.

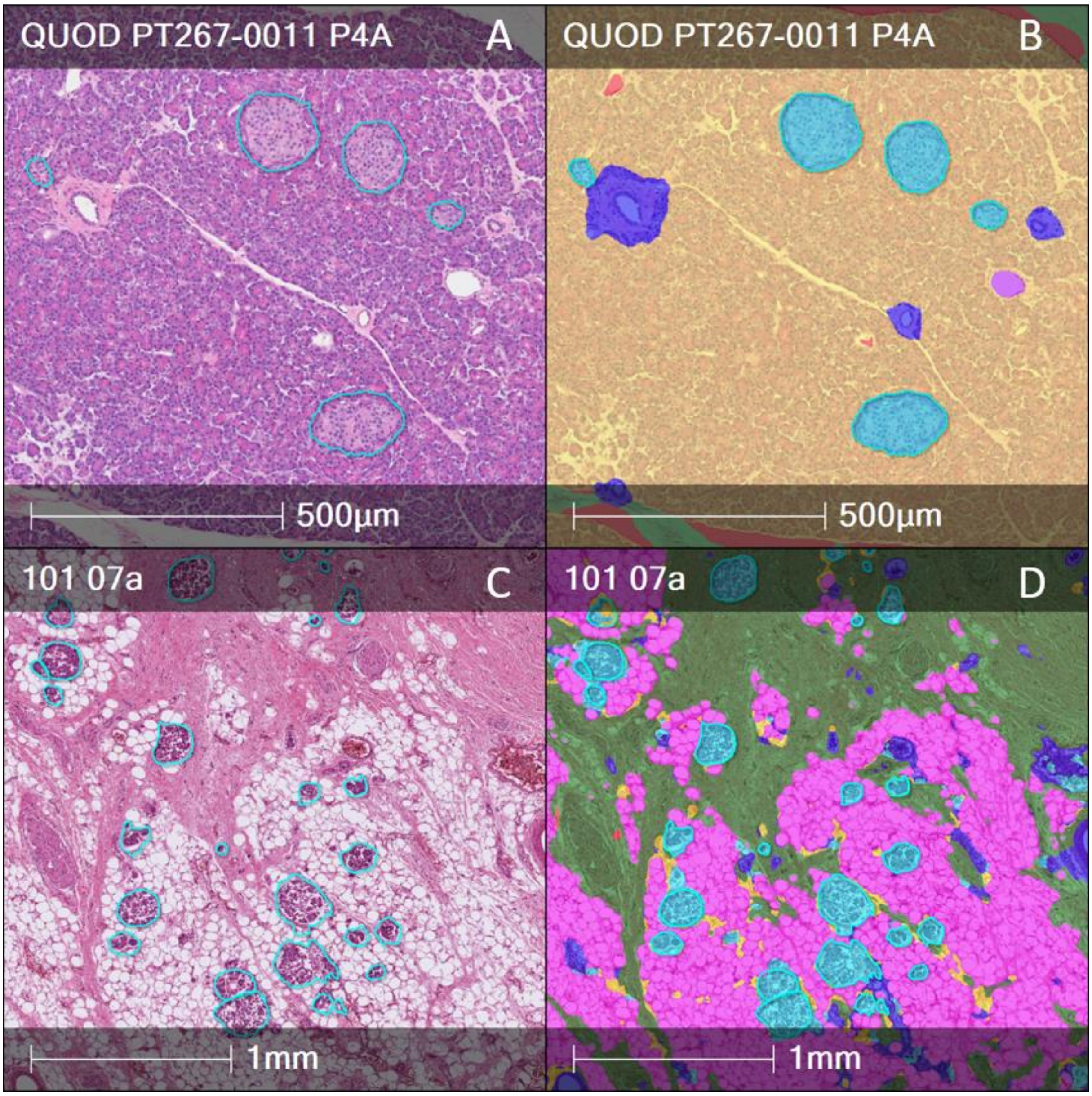

ESM Fig. 10: AI classifier illustration of H&E area quantification in a 27 years' old male QUOD control (Case number: 29) (A and B) and a 27 years' old male CF donor (Case number: CF28) (C and D). Tissue classifier colours: islets (cyan); ducts (blue); fat (pink); acinar (yellow); background (red); fibrosis (green).

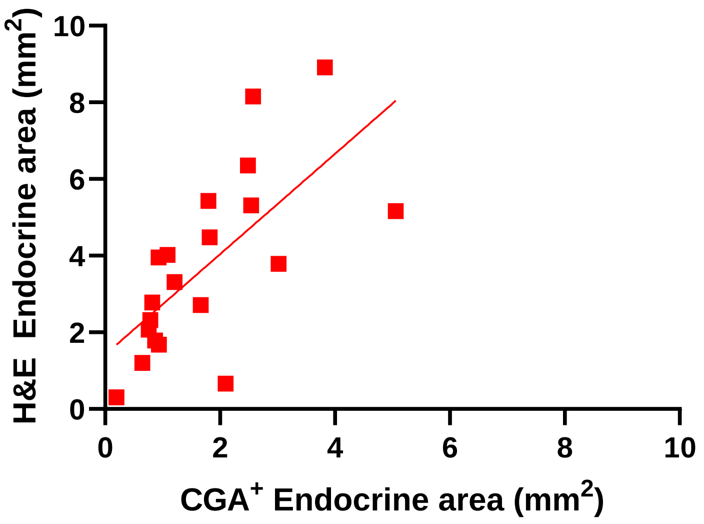

ESM Fig. 11: Block-based AI assessment of endocrine area using H&E staining and CGA staining of CF pancreata of different patterns. Pearson's correlation plot to show correlation between classified endocrine area in CF tissue (20 blocks) using H&E staining and CGA staining to check comparability, r^2= 0.465, p=0.0009.

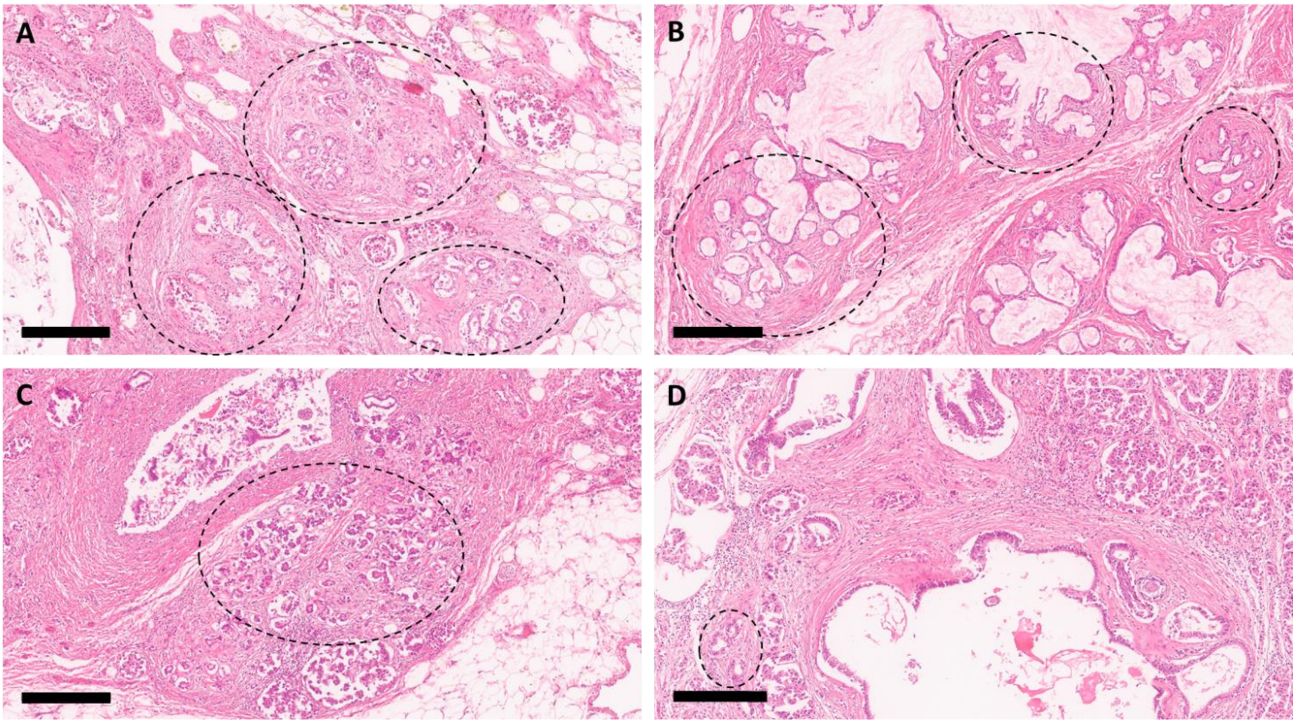

ESM Fig. 12: H&E staining of CF Case number 28 (A), 22 (B), 27 (C), and 29 (D). Dashed circles show small residual ducts surrounded by fibrosis. Scale bars 300 µm.

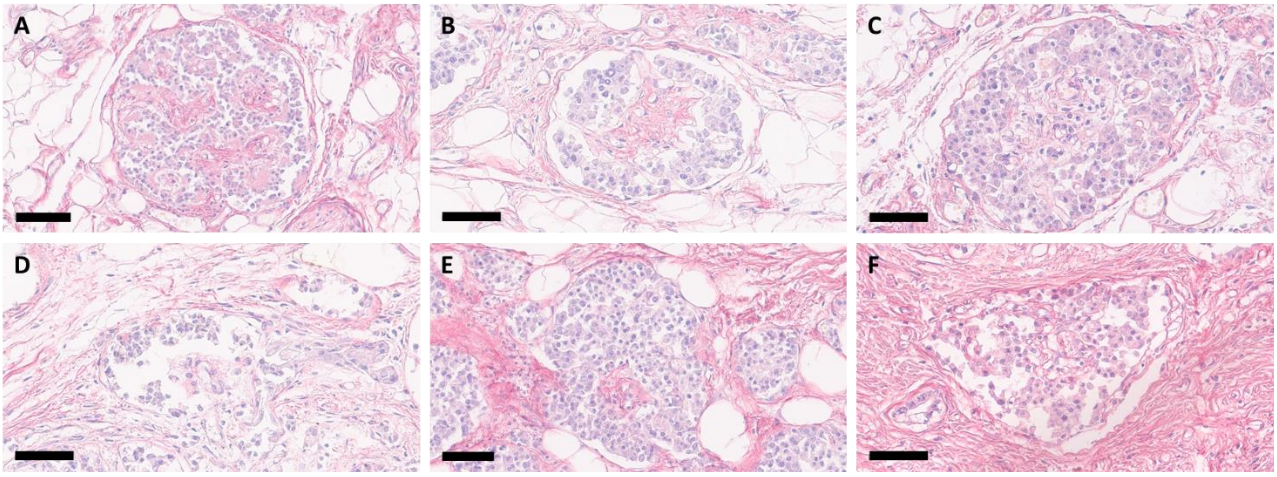

ESM Fig. 13: SRFG staining of CF Case number 22 (A-C), 28 (D), 29 (E) and 27 (F) showing intra-islet and peri-islet fibrosis. Scale bars: 60 µm (B, C, D, F), 70 µm (E), 80 µm (A).

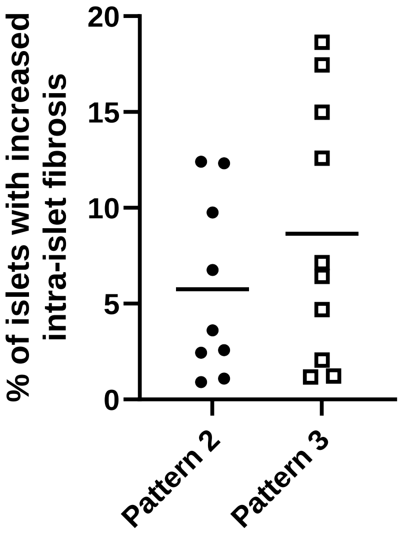

ESM Fig. 14: Manual quantification of the percentage of islets with significant fibrosis extending beyond the intra-islet perivascular region in CF Pattern 2 and CF Pattern 3. No significant difference between the patterns was observed (p=0.3015). All islets which could be identified on the SRFG stained sections were assessed for increased intra-islet fibrosis defined as increased collagen stain compared to collagen stain around vasculature. Sections of CF Pattern 1 could not be analysed with this method due to difficulties in identifying the islets without an endocrine staining.

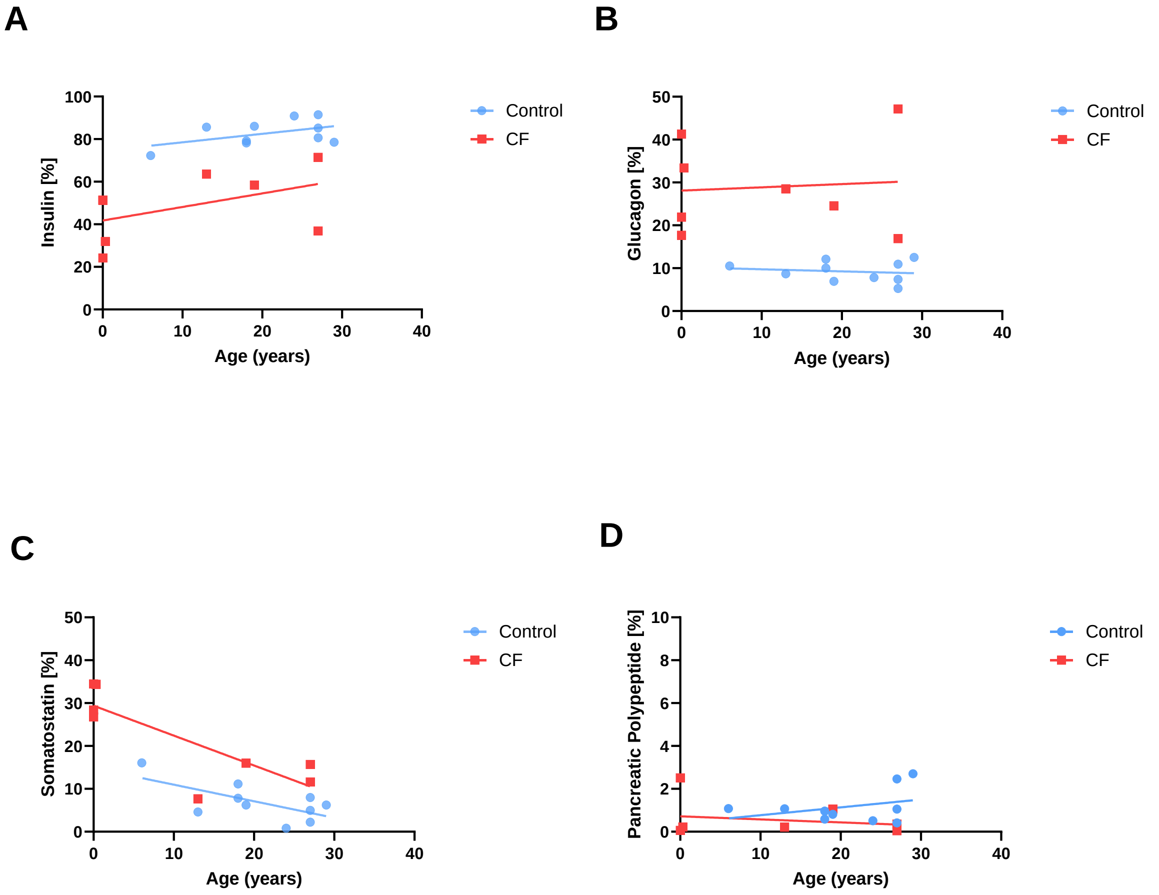

ESM Fig. 15: Islet hormone distribution in CF and non-CF pancreata. Percentage insulin (A), glucagon (B), somatostatin (C) and pancreatic polypeptide (D) PA vs donor age in control (blue) and CF (red) donors. For donors with multiple blocks mean values were used.

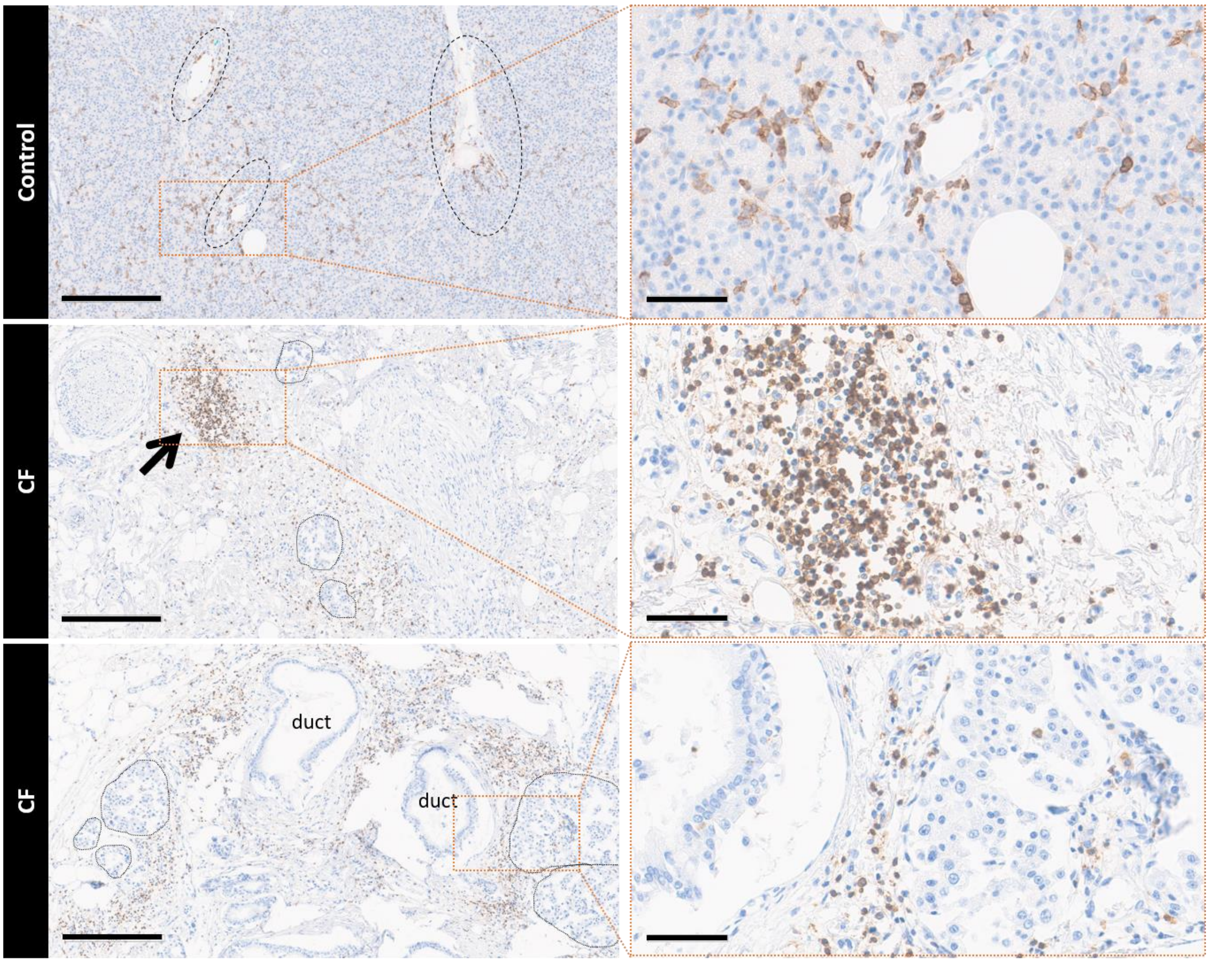

ESM Fig. 16: Leucocyte distribution in control and CF pancreata. Example pictures of CD45 immunostaining (brown) of control (top panel, donor Number 50) and CF pancreata (middle and bottom panel; donor Number 29). Dashed lines highlight vessels, solid lines highlight islets, arrow indicates inflammatory focus. Scale bars 300 µm left column, 60 µm right column.

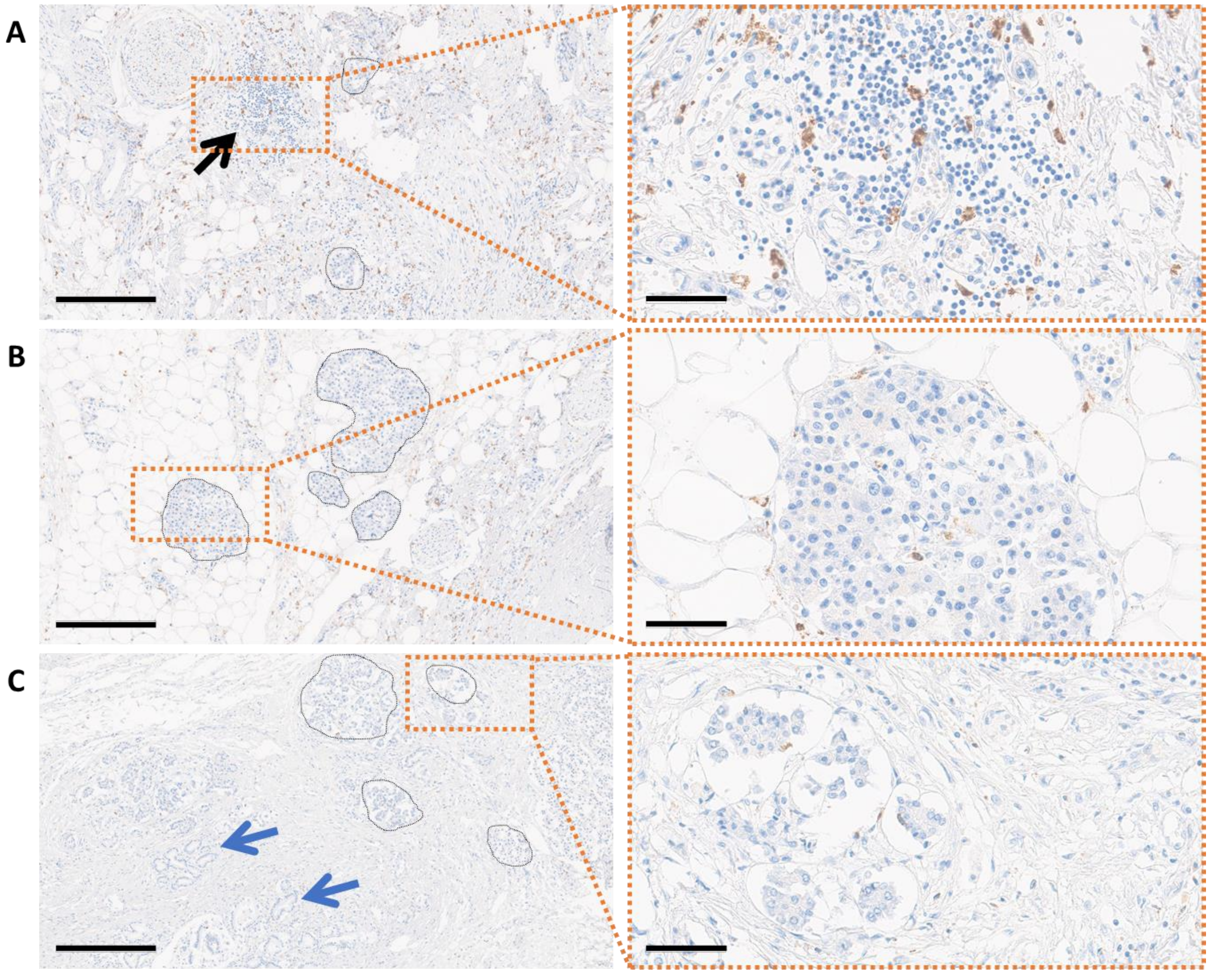

ESM Fig. 17: CF pancreas stained for CD68 (brown). A, B: different blocks from donor Number 29. C: donor Number 27. Scale bars: 300 µm left column, 60 µm right column. A shows a corresponding area to the CD45 staining in Supplementary Figure S15 D2 highlighting macrophages in and around a focus of lymphocytic inflammation. B and C show peri- and intra-islet macrophages. Blue arrows in C indicate areas of small ducts. Solid lines highlight islets.

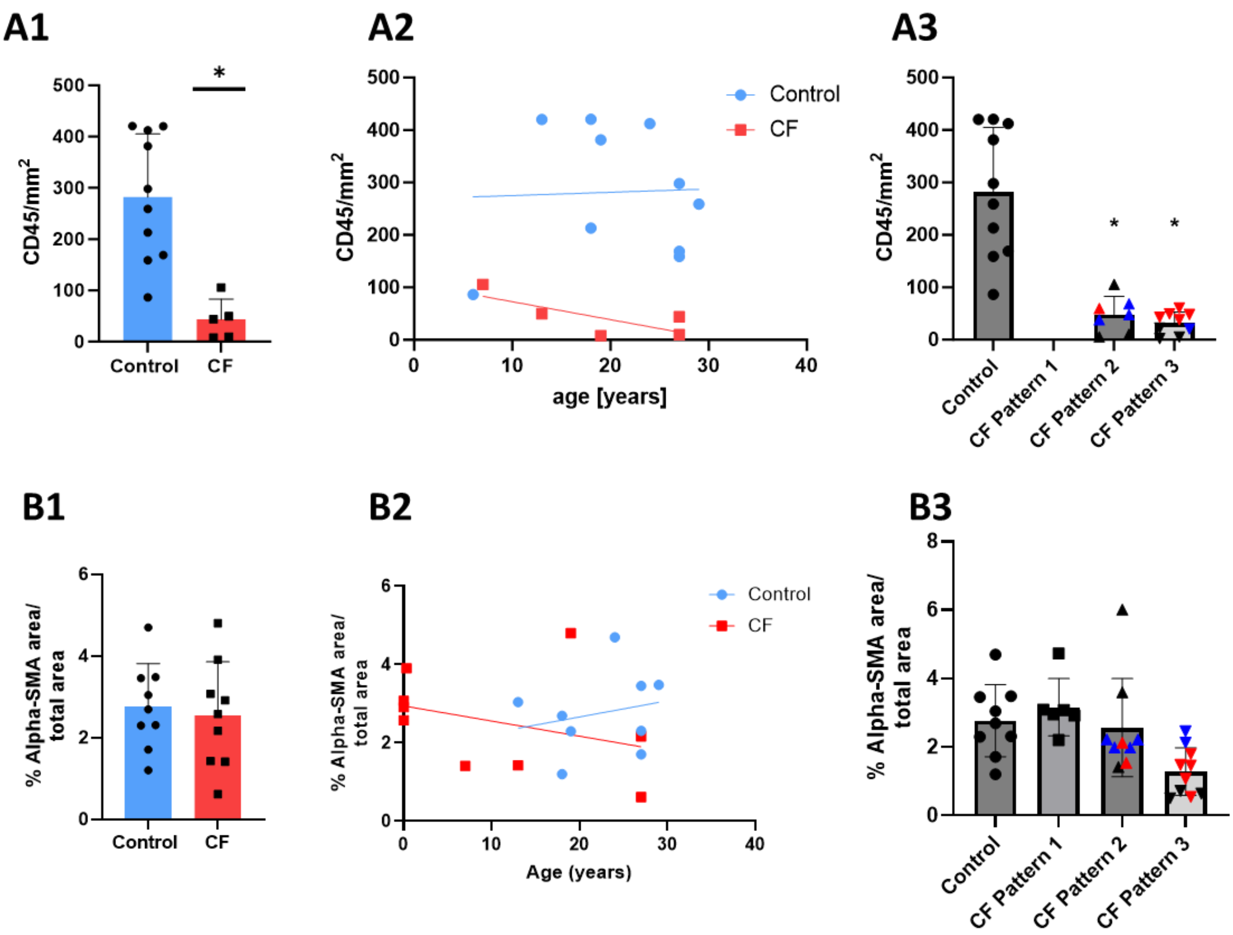

ESM Fig. 18: AI augmented quantification of CD45 counts (A1-A3) and alpha-SMA stained area (B1-B3) in whole tissue sections of CF and non-CF pancreata. The Indica Labs HALO HighPlex BF module was used to enumerate CD45 positively stained cells and quantify alpha-SMA proportional area. (A1, B1) Control (blue) and CF (red) donors were compared by Student's T-test. (A2, B2) Donor-based analysis vs age in control (blue circles) and CF (red squares) donors. (A3, B3) Block-based analysis of control vs CF Pattern 1, 2, and 3. Linear mixed effect model (LMEM) was used with histological CF pattern set as fixed effect, donor as random effect and Bonferroni post hoc test was utilised for statistical analysis. Graphs show mean±SD. $p<0.05$ was considered statistically significant. (*) indicates significant difference compared to control. Data points coloured red represent Case 22 and points coloured blue represent Case 29, both donors having multiple blocks of different patterns. (A1, A2) control n=10 donors, CF n=5 donors, (A3) control n=10 blocks, CF Pattern 1 n=0 blocks, CF Pattern 2 n=7 blocks, CF Pattern 3 n=9 blocks. (B1, B2) control n=9 donors, CF n= 9 donors, (B3) control n=9 blocks, CF Pattern 1 n=6 blocks, CF Pattern 2 n=9 blocks, CF Pattern 3 n=10 blocks.

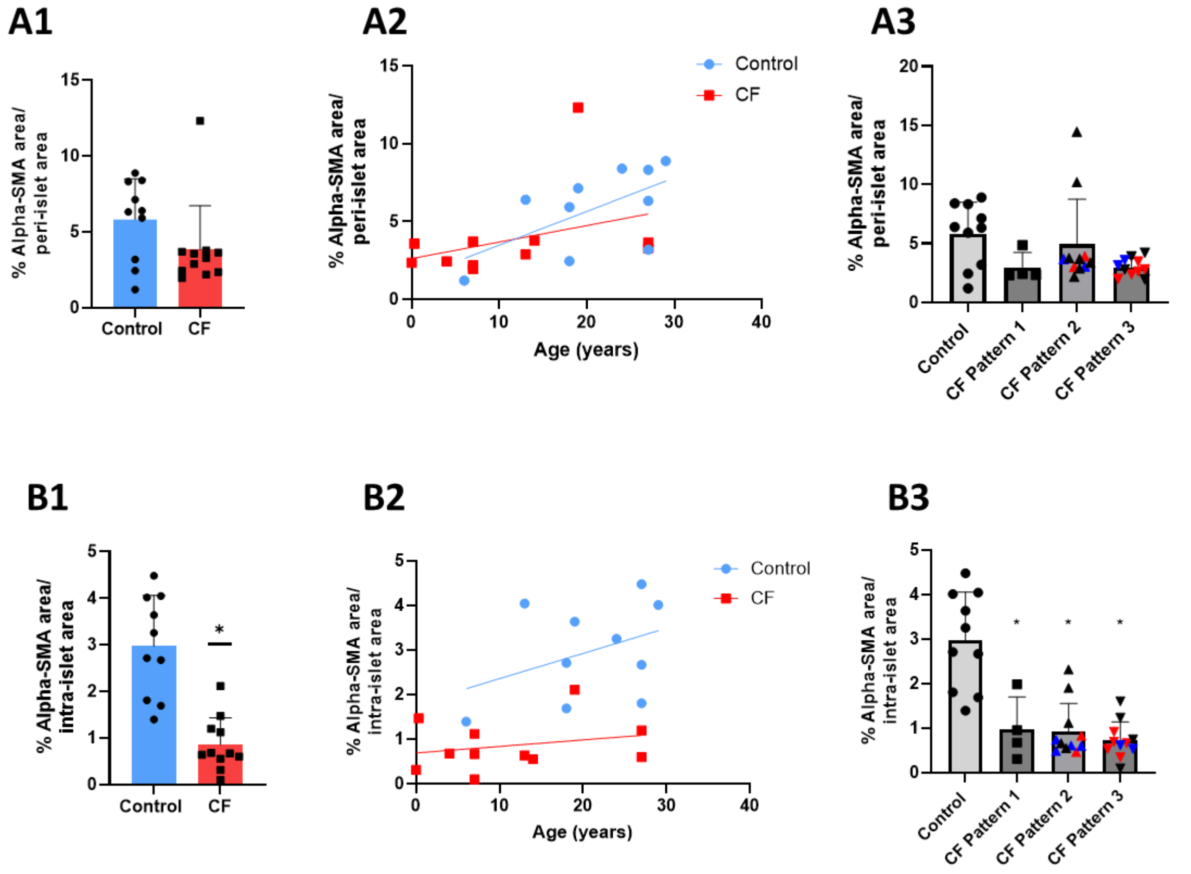

ESM Fig. 19: AI augmented quantification of peri-islet (A1-A3) and intra-islet (B1-B3) alpha-SMA stained area in CF and control donors. The Indica Labs HighPlex BF module was used to quantify alpha-SMA proportional area within previous classified peri- and intra-islet regions. (A1, B1) Control (blue) and CF (red) donors were compared by Student's T-test. (A2, B2) Donor-based analysis vs age in control (blue circles) and CF (red squares) donors. (A3, B3) Block-based analysis of control vs CF Pattern 1, 2, and 3. Linear mixed effect model (LMEM) was used with histological CF pattern set as fixed effect, donor as random effect and Bonferroni post hoc test was utilised for statistical analysis. Graphs show mean±SD. $p<0.05$ was considered statistically significant. (*) indicates significant difference compared to control. Data points coloured red represent Case 22 and points coloured blue represent Case 29, both donors have multiple blocks of different patterns. (A1, A2, B1, B2) control n=10 donors, CF n=11 donors. (A3, B3) control n=10 blocks, CF Pattern 1 n=4 blocks, CF Pattern 2 n=11 blocks, CF Pattern 3 n=11 blocks.
